# 3D bioprinting of collagen-based high-resolution internally perfusable scaffolds for engineering fully biologic tissue systems

**DOI:** 10.1126/sciadv.adu5905

**Published:** 2025-04-23

**Authors:** Daniel J. Shiwarski, Andrew R. Hudson, Joshua W. Tashman, Ezgi Bakirci, Samuel Moss, Brian D. Coffin, Adam W. Feinberg

**Affiliations:** ^1^ Department of Biomedical Engineering, Carnegie Mellon University, Pittsburgh, PA 15213, USA.; ^2^ Department of Bioengineering, University of Pittsburgh, Pittsburgh, PA 15213, USA.; ^3^ Pittsburgh, Heart, Lung, and Blood Vascular Medicine Institute, University of Pittsburgh, Pittsburgh, PA 15213, USA.; ^4^ Department of Medicine, University of Pittsburgh School of Medicine, Pittsburgh, PA 15213, USA.; ^5^ Department of Materials Science and Engineering, Carnegie Mellon University, Pittsburgh, PA 15213, USA.

## Abstract

Organ-on-a-chip and microfluidic systems have improved the translational relevance of in vitro systems; however, current manufacturing approaches impart limitations on materials selection, non-native mechanical properties, geometric complexity, and cell-driven remodeling into functional tissues. Here, we three-dimensionally (3D) bioprint extracellular matrix (ECM) and cells into collagen-based high-resolution internally perfusable scaffolds (CHIPS) that integrate with a vascular and perfusion organ-on-a-chip reactor (VAPOR) to form a complete tissue engineering platform. We improve the fidelity of freeform reversible embedding of suspended hydrogels (FRESH) bioprinting to produce a range of CHIPS designs fabricated in a one-step process. CHIPS exhibit size-dependent permeability of perfused molecules into the surrounding scaffold to support cell viability and migration. Lastly, we implemented multi-material bioprinting to control 3D spatial patterning, ECM composition, cellularization, and material properties to create a glucose-responsive, insulin-secreting pancreatic-like CHIPS with vascular endothelial cadherin^+^ vascular-like networks. Together, CHIPS and VAPOR form a platform technology toward engineering full organ-scale function for disease modeling and cell replacement therapy.

## Teaser

Bioreactor maturation of high-resolution FRESH 3D-bioprinted scaffolds form vascularized centimeter-scale functional tissues.

## INTRODUCTION

The cardiovascular system is a developmental requirement for all vertebrates because it transports oxygen and nutrients to tissues and organs and removes metabolic waste. This need for perfusable fluidic networks has posed a major challenge in the bioengineering field, often resulting in the engineering of tissue scaffolds and devices that either constrain thickness to the limits of passive nutrient diffusion (~200 μm) or result in necrotic cores within larger constructs due to hypoxia (*1*, *2*). A key approach toward addressing this has been the development of microfluidic and organ-on-a-chip systems to model vascular flow and biological processes, including lung-on-a-chip, miniature cardiac pumps, multi-organ systems, and three-dimensional (3D) vascular networks (*3*–*6*). These microphysiological systems have proved to be useful platforms for studying vasculogenesis and angiogenesis, as well as modeling human diseases and pharmaceutical responses that cannot be studied in patients (*7*). However, the materials for microfluidic chip production have traditionally been plastics and elastomers such as polydimethylsiloxane (PDMS) (*8*, *9*) that are orders-of-magnitude stiffer than native tissue, may absorb lipophilic biomolecules out of the media, require photolithography and a clean room to manufacture, cannot be remodeled by cells into more complex structures, and can only be used in vitro (*10*, *11*). Building perfusable scaffolds from hydrogel-based materials has been recognized as a means to overcome these challenges and better mimic the native extracellular matrix (ECM) (*8*, *12*, *13*). High-resolution 3D printing of microfluidics using photoresins such as polyacrylates (*14*) and biocompatible hydrogels like poly(ethylene glycol) (PEG)–diacrylate and gelatin methacryloyl (*6*) address some of these aspects but are typically limited to a single material and must still be cellularized by perfusion seeding after device fabrication (*15*). Similarly, extrusion 3D bioprinting of vascular-like networks using sacrificial polymers produces perfusable tissue constructs, but the spatial resolution and design complexity are limited (*16*, *17*). Thus, while multiple fabrication technologies have been developed, limitations remain in terms of spatially patterning cells, hydrogels, ECM, and other components in 3D and thus the structural complexity and function of the model systems that can be built.

Here, we report the direct 3D bioprinting of collagen-based hydrogels, ECM, and cells into fully biologic perfusable scaffolds with high-fidelity control of structure and composition. To do this, we leverage the versatile fabrication capabilities of freeform reversible embedding of suspended hydrogels (FRESH) (*18*, *19*) to merge the top-down engineering of perfusable hydrogel scaffolds with bottom-up, cell-driven self-assembly to bridge the macro (>1 mm) and micro (<1 mm) length scales. Specifically, we FRESH 3D bioprint cells, collagen type I, fibrin, and other ECM components and growth factors into complex 3D constructs termed collagen-based high-resolution internally perfusable scaffolds (CHIPS). As the primary structural protein in the body (*20*), collagen type I is an ideal material to serve as the structural bioink for CHIPS by providing mechanical strength as well as defining vascular and tissue compartments. Further, to perfuse these relatively soft collagen-based scaffolds, we developed a novel bioreactor system termed vasculature and perfusion organ-on-a-chip reactor (VAPOR) to dynamically culture CHIPS. The ability to rapidly iterate VAPOR and CHIPS design in computer-aided design (CAD) and immediately FRESH print it results in a highly modular tissue engineering platform. Here, we demonstrate this by fabricating and validating more than 10 different CHIPS designs, including glucose-responsive, insulin-secreting pancreatic-like CHIPS that form capillary-like networks under perfusion culture. Together, the microfluidic CHIPS and VAPOR bioreactor form an integrated system that can recreate established microfluidic capabilities while enabling a new class of constructs that combine cells with ECM proteins such as collagen and fibrin into functional, centimeter-scale tissue systems.

## RESULTS

### Design, fabrication, and metrology assessment of 3D-bioprinted CHIPS

**Fig. 1.**
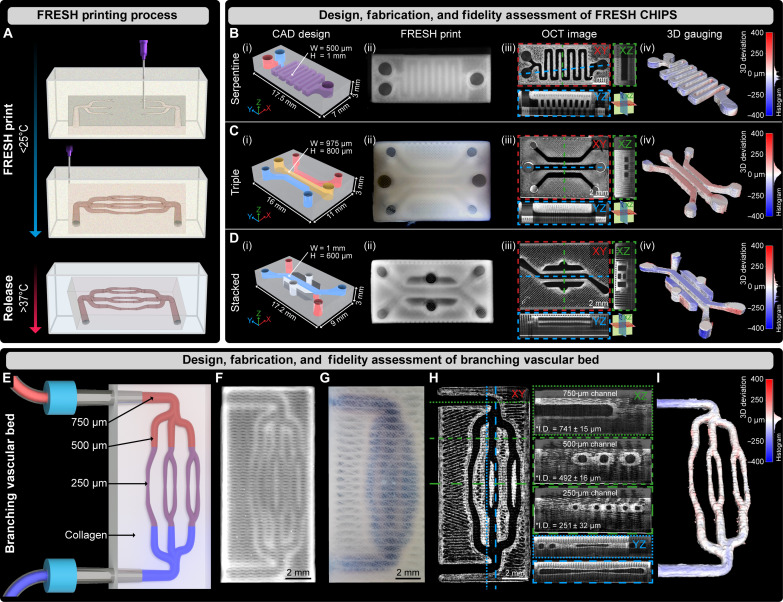
Fabrication, 3D gauging, and perfusion of FRESH-printed CHIPS. (**A**) Schematic of the FRESH bioprinting and release process. (**B** to **D**) CAD design (i); FRESH printing using a collagen bioink (ii); volumetric OCT imaging revealing high-resolution control over collagen extrusion and negative space resulting in patent lumens (dark regions) (iii); and 3D gauging of the negative space luminal region with histogram of the over and under print error for quantitative fidelity assessment of serpentine, triple, and stacked channel internal network designs (iv). (**E**) A multi-scale vascular bed design with open lumens from 1 mm to 250 μm. (**F**) Multi-scale vascular bed FRESH printed from collagen I. (**G**) Perfusion of blue dye through the multi-scale vascular scaffold. (**H**) Volumetric OCT imaging and cross-sectional analysis demonstrating lumen patency and circular fidelity of the internal fluidic network. (**I**) 3D gauging of the multi-scale vascular lumen volume reveals high-fidelity printing with average deviations <11 μm.

CHIPS are FRESH printed via the direct extrusion of a biomaterial into a thermo-reversible support bath consisting of a gelatin microparticle slurry. The support bath performs two critical functions by (i) immobilizing an embedded bioink filament where it is deposited during the printing process due to its Bingham-plastic rheology and (ii) triggering the rapid gelation or curing of the embedded filament (Fig. 1A) (*18*, *19*). For collagen-based bioinks (spanning a 12- to 35-mg/ml concentration range found in soft tissue) (*21*), the aqueous fluid phase of the support bath is pH-buffered to rapidly neutralize acidified collagen and drive the self-assembly of a fibrillar network. Upon print completion, the support bath is removed by raising the temperature to 37°C, which melts the gelatin, allowing for nondestructive print retrieval. While we have used FRESH to produce 3D structures in the past (*19*), improved tolerancing of our extruded material was required to successfully produce single or multi-material CHIPS with reliably perfusable internal channels.

In the context of tissue engineering, vascularized tissues can be viewed as being composed of precisely patterned positive (cells, ECM, etc.) and negative (vascular, lymphatic, etc.) space. As an additive manufacturing process, 3D printing facilitates the patterning of positive and negative space in a layer-by-layer manner via the accurate placement or curing of material. Our group has demonstrated that by altering the needle inner diameter (ID) used during FRESH printing, we can tune the single-filament resolution to 20 μm (*19*); however, in practice, the filament size only defines part of the total resolution for a given object. As CHIPS resolution in total depends on patterning the negative and positive space, we sought to measure both feature types to define print fidelity for reliable internal perfusability. Our initial goal was to refine our FRESH printing process to improve filament reproducibility (deviation from intended diameter), mechanical accuracy (filament positioning in XY), and layer-by-layer repeatability (layer alignment in Z) throughout a range of geometric complexity.

Using CAD, we created three centimeter-scale microfluidic devices inspired by organ-on-a-chip designs that were originally fabricated using photolithography or light-based 3D printing. Specifically, we fabricated CHIPS with (i) a serpentine mixing network similar to designs reported by Whitesides and coworkers (*22*) with internal channel dimensions of 500 μm wide by 1000 μm tall (Fig. 1, Bi), (ii) a triple channel design separated by narrow 380-μm collagen walls resembling Kamm and coworkers’ work (*23*) (Fig. 1, Ci), and (iii) a stacked channel device similar to a lung-on-chip design by Ingber and coworkers (*3*) with multiple channels separated by 400 μm in Z using a one-step FRESH printing process (Fig. 1, Di). Each model was imported into open-source slicing software to generate 3D printer machine pathing known as G-code. The G-code previews provide a visualization of the type of biomaterial filaments extruded (external perimeter, internal perimeter, infill, and surface) in a layer-by-layer manner to create the final 3D structure (fig. S1, A to I). To ensure perfusability within CHIPS, all positive and negative features within the G-code must accurately and precisely match the intended CAD geometry since the minimum perfusable channel diameter is governed by patterning negative space and not solely dependent on the fidelity of the extruded filament. While this may seem like an obvious constraint, for CHIPS, only 35% of the scaffold volume is made of positively extruded infill, and the remaining 65% volume is left as negative space.

Optical imaging of the serpentine, triple, and stacked CHIPS confirmed successful FRESH printing using collagen-I bioink. The translucent nature of the FRESH-printed collagen enables visualization of the internally perfusable networks under stereomicroscopy as darker regions outlined by the opaque collagen perimeters [Fig. 1, B (ii) to D (ii)]. In addition, to obtain detailed structural data throughout the 3D volume of the CHIPS, we performed volumetric imaging using optical coherence tomography (OCT). This noninvasive imaging modality is ideal for CHIPS since the differences in refractive index (RI) between the extruded collagen (RI = 1.41) and the fluid-filled void space (RI = 1.33) creates a high contrast image. For each CHIPS design, XY, XZ, and YZ cross-sectional images from the OCT volumes highlight the exact patterning of the extruded collagen filaments (white) and the precision of the negative space (black) to produce internally patent channels [Fig. 1, B (iii) to D (iii)].

Next, we confirmed proper mechanical calibration based on five parameters measured from OCT to define high-resolution extruded scaffold fidelity: filament diameter_X,_ filament diameter_Y_, infill spacing_X,_ infill spacing_Y_, and layer straightness_XZ_. A region of interest (ROI) within the Stacked CHIPS OCT XY cross section corresponding to the G-code for layer 59’s infill features (fig. S1G) was chosen for analysis because of its defined rectilinear structure and ability to resolve both individual filaments and negative space (fig. S1, J and K). The average filament diameter printed in both the X and Y motion directions was quantified as 105 ± 9 μm. This slight filament deviation beyond 90 μm is expected because of filament compression that occurs during layer-layer stacking and provides beneficial mechanical strength and improved layer adhesion compared to under-extrusion. The infill spacing in X and Y (a measurement of 3D printer mechanical accuracy and repeatability) was highly repeatable with an average deviation from the expected position of <10 μm and a perpendicularity of 89.5° ± 1.2° (*n* = 30 intersections). Lastly, since 3D bioprinting is a layer-by-layer additive manufacturing technique, as individual layers are added, the alignment between each layer can be measured in Z to determine vertical straightness as layers are stacked (fig. S1L). Using an XZ cross section from the OCT images, we obtained an average layer straightness for the stacked extruded filaments to be within 6 μm of the true vertical expectation (fig. S1M). These and other measurements (fig. S1N) are essential to confirm that CHIPS will faithfully recapitulate the designed CAD model when printed and produce high-fidelity positive features as well as defect-free negative space to reliably create perfusable small diameter channels.

The final step in validating the fabrication of CHIPS was to determine the printed fidelity of the internally perfusable channels. To isolate the perfusable networks from the bulk scaffold, an inverse model of the CHIPS was created and compared to a segmented 3D volume based on OCT images for quantitative gauging using a 3D point cloud–based registration process. This process allows for a true volumetric measurement of the deviation between the channels printed within CHIPS and the original CAD model. For serpentine, triple, and stacked CHIPS, the average overprint and underprint root mean squared (RMS) error for the entire volume was less than 20 μm (histograms for model-specific RMS deviation are shown) [Fig. 1, B (iv) to D (iv)]. Together, these parameters define high-fidelity printing for both the extruded features and negative space pushing the bounds of our 3D printer’s mechanical motion control system. In addition, these FRESH-printed CHIPS set a fabrication standard for extrusion-based bioprinting as all error measured is approaching both the imaging resolution limit of the OCT system (16-μm voxel in XY) and the mechanical performance of our bioprinter (± 8 μm).

To test the capabilities of our improved high-fidelity printing process and highlight CHIPS design flexibility, we designed a vascular-like network with circular lumens branching from 750-μm ID inlets down to 250-μm ID (Fig. 1E). This design was FRESH printed (Fig. 1F and movie S1) and manually perfused with a blue dye to demonstrate network patency and integrity of the channel walls (Fig. 1G). Quantitative OCT–based image analysis confirmed patent channels, clearly defined individual collagen infill features, and circular channel cross sections with average diameters for the branched segments of 741 ± 15, 492 ± 16, and 251 ± 32 μm, respectively (Fig. 1H). Moreover, 3D gauging comparing the CAD design of the perfusable network to the OCT scan data of the negative space within the printed CHIPS measured an average overprint and underprint RMS error of 11 μm (Fig. 1I), showing that independent of the geometry or network design, FRESH-printed CHIPS have excellent fidelity for channels of both square (Fig. 1, B to D) and circular (Fig. 1, E to I) cross sections. Moreover, these examples demonstrate the highest fidelity of collagen-based bioprinting achieved using any hardware or software platform to produce a broad range of CHIPS designs that can be FRESH 3D bioprinted in a one-step fabrication process.

### Perfusion of CHIPS using VAPOR bioreactors

**Fig. 2.**
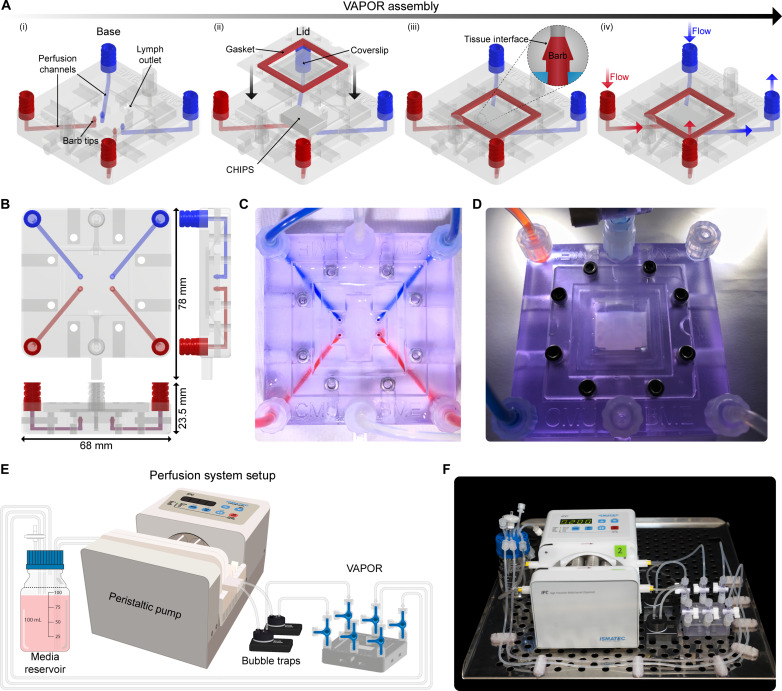
Design and implementation of the VAPOR bioreactor platform. (**A**) CAD model and schematic of the (i) VAPOR base, (ii) lid and gasket assembly, (iii) CHIPS and VAPOR barb interface for watertight seal, and (iv) dual independent flow paths through VAPOR and CHIPS. (**B**) Dimensions of the assembled VAPOR system. (**C**) Image of VAPOR perfused with red and blue dye to visualize the internal fluidic networks. (**D**) Fully assembled VAPOR and inserted serpentine CHIPS before perfusion. (**E**) Schematic of the perfusion setup used in VAPOR. (**F**) Image of the assembled VAPOR platform and perfusion system before installation into a cell culture incubator.

Unlike organ-on-a-chip systems made from PDMS, thermoplastics, and photoresins, which are rigid enough to support direct inlet and outlet cannulation for perfusion (*13*), the softer FRESH 3D-bioprinted CHIPS required the engineering of a new perfusion approach to ensure flow and prevent leaking when pressurized. To address this challenge, we developed the VAPOR bioreactor consisting of two components: (i) a main body 3D printed from ultraviolet (UV)–curable biocompatible resin with integrated Luer lock connection fittings and internal fluidic channels and (ii) a removable lid containing a silicone gasket and glass imaging window (Fig. 2, A to D). The internal fluidic channels of VAPOR terminate in barbed fittings, a critical design feature, as all CHIPS for VAPOR have the negative space of the barb incorporated into the inlet and outlet geometry to form an interlocking connection when assembled (Fig. 2A). In addition, VAPOR includes a lymph-like fluidic path that provides an outlet from the main chamber to a separate Luer lock connection which (i) relieves pressure spikes during initial assembly, (ii) provides a bypass for fluid if there is a main flow blockage, and (iii) allows for recirculation of interstitial fluid that diffuses out of the CHIPS (Fig. 2Ai). A peristaltic pump, media reservoir, and bubble trap complete the perfusion circuit (Fig. 2E), and the entire setup is placed in a cell culture incubator for cellular perfusion studies (Fig. 2F). Most perfusion studies were performed at a flow rate of 100 μl/min, but we validated that CHIPS could support flow rates of at least 1000 μl/min. On a basis of a channel 500 μm high, 1000 μm wide, and 8 mm long and perfused with EGM2 media at 37°C with a viscosity of 0.78 cP (*24*), this results in a wall shear stress of ~3 dyne/cm^2^, which is within the range of 1 to 10 dyne/cm^2^ reported for large veins and small arteries (*25*). Since both the CHIPS and VAPOR are 3D printed, the overall dimensions and placement of inlets and outlets, as well as the internal perfusable network are easily modified and iterated upon to meet specific experimental needs.

**Fig. 3.**
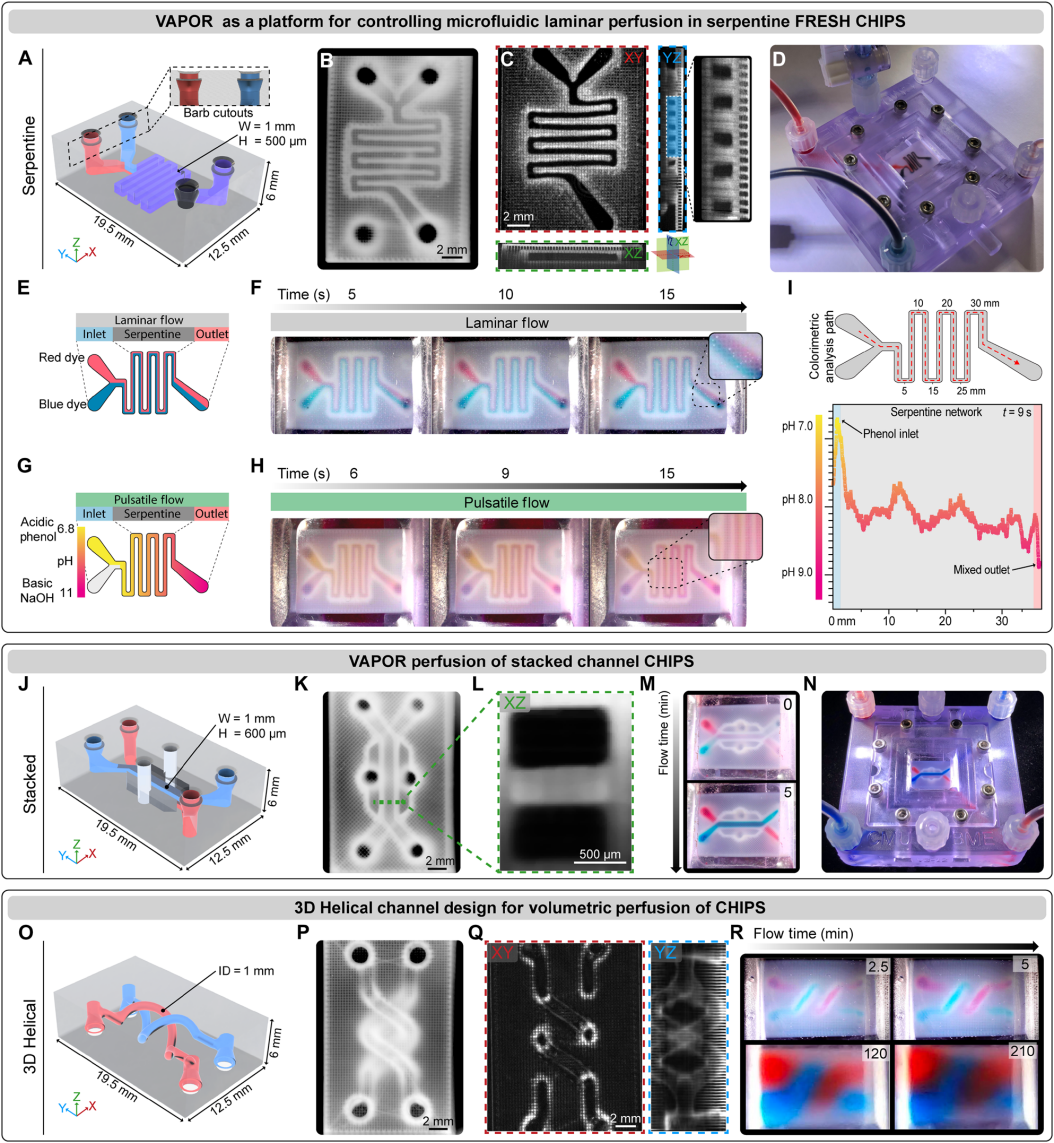
VAPOR perfusion of FRESH CHIPS. (**A**) CAD design of a dual inlet single outlet serpentine network CHIPS with barb-shaped cutouts for mating to the VAPOR inlets and outlets. (**B**) Stereomicroscope image of a FRESH-printed serpentine CHIPS. (**C**) OCT volumetric imaging and cross-sectional analysis revealing patent 500-μm lumens (dark regions). (**D**) VAPOR perfusion of serpentine CHIPS with red and black dye. (**E**) Graphic illustration of a laminar flow perfusion experiment to demonstrate balanced laminar flow of two fluids within a serpentine CHIPS. (**F**) Serpentine CHIPS perfused with a red and blue dye demonstrating laminar perfusion and minimal interfacial mixing. (**G**) Graphic illustration of a pulsatile flow perfusion experiment to demonstrate fluidic mixing within a serpentine network using an acidic phenol red indicator dye and basic NaOH solution. (**H**) Pulsatile flow in serpentine CHIPS produces a mixing gradient along the fluidic path. (**I**) Quantitative colorimetric analysis of the pH-based mixing profile achieved within the serpentine channel as the phenol red is neutralized. (**J**) CAD design and adaptation of stacked channel CHIPS for interfacing with VAPOR. (**K**) Stereomicroscope image of a FRESH-printed stacked CHIPS. (**L**) An OCT XZ cross-sectional image of the stacked channels highlighting the fidelity and resolution achieved via FRESH bioprinting collagen. (**M**) Images showing initiation of stacked CHIPS perfusion of red and blue dye in VAPOR. (**N**) Overview image of the perfused stacked CHIPS within VAPOR. (**O**) CAD model of the 3D helical CHIPS network. (**P**) Stereomicroscope image of a FRESH-printed 3D helical CHIPS. (**Q**) OCT volumetric imaging and cross-sectional analysis showing patent circular lumens (XY) and a YZ projection image to view the full helical network. (**R**) VAPOR perfusion of 3D helical CHIPS for 210 min resulting in complete volumetric diffusion of low molecular weight dyes (red and blue) throughout the CHIPS.

To assess perfusion integration, a CHIPS design containing an internal serpentine channel 500 μm wide and 1 mm tall (Fig. 3A) was FRESH printed (Fig. 3B and fig. S2, A to D), verified for lumen patency by OCT (Fig. 3C and fig. S2E), placed in the VAPOR bioreactor, and then perfused (Fig. 3D and movie S2). Our first goal was to achieve laminar flow within the CHIPS device by simultaneously perfusing red and blue dyed solutions into the inlets (Fig. 3E, Reynolds number, *Re* = 0.37). Video time-lapse imaging revealed two distinct parallel dye streams throughout the serpentine channel with mixing only at the dye interface due to the relatively short path length (Fig. 3F and movie S2). Next, we performed a pulsatile perfusion experiment to drive mixing between an acidic phenol red solution (starting as a visible yellow solution, pH 6.8) and a clear basic NaOH solution (pH 11) (Fig. 3G). Pulsatile flow resulted in rapid mixing and a pH-dependent color change from yellow to magenta along the length of the serpentine path (Fig. 3H and movie S2). Colorimetric analysis of the phenol red pH indicator confirmed a pH change from 6.8 to 9 along the channel, with a wave-like pH profile driven by changes in the fluid dynamics (*26*) that occur at turns within serpentine networks (Fig. 3I).

With the VAPOR platform fully functional, we sought to further demonstrate the diversity in design space of CHIPS by fabricating and perfusing multilayered and helical channel designs. A multilayered CHIPS with stacked channels based on the established lung-on-a-chip model (Fig. 1D) (*3*) was designed with two 1000 μm–by–600 μm channels separated in the *z* axis by a 400-μm collagen barrier (Fig. 3, J to L; fig. S2F; and movie S3). Perfusion of red and blue dye within the stacked channels resulted in two independent fluid streams separated by the collagen barrier (Fig. 3, M and N, and movie S3). Next, we designed a CHIPS containing two circular channels in a double helix configuration to highlight volumetric 3D patterning of internally perfusable networks (Fig. 3O). This was FRESH printed with high fidelity (Fig. 3, P and Q; fig. S2G; and movie S3) and perfused to demonstrate time-dependent diffusion of small (~700 Da) red and blue dyes into the bulk collagen scaffold over 3.5 hours (Fig. 3R and movie S3). The diffusion observed throughout the helical CHIPS is due to a combination of diffusion through the collagen walls of the channels and diffusion through the internal open lattice structure defined by the percentage of positive infill (35%) and negative space (65%) during the G-code slicing.

### Molecular weight–dependent diffusion within perfused CHIPS

**Fig. 4.**
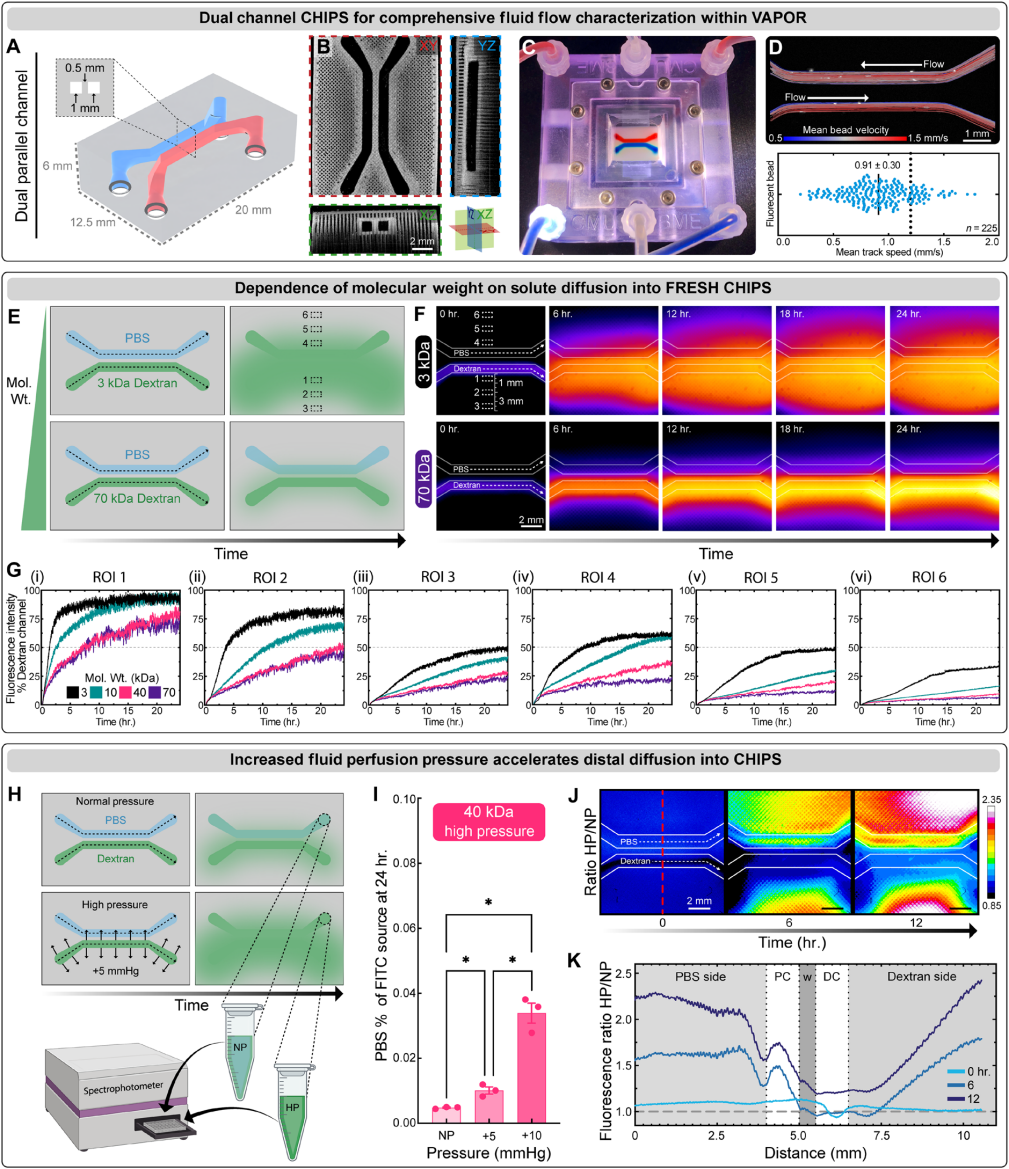
Characterization of diffusivity within FRESH CHIPS. (**A**) A CAD model of dual parallel channel CHIPS with the channels separated by a collagen wall. (**B**) OCT cross-sectional views of dual parallel channel CHIPS. (**C**) VAPOR perfusion of dual parallel channel CHIPS with red and blue dye. (**D**) Perfusion and tracking of fluorescent microbeads to measure velocity profiles and mean bead speed compared to the theoretical estimation (dotted line). (**E**) A schematic demonstrating the perfusion of dual parallel channel CHIPS where one channel is always perfused with PBS while the other channel is perfused with different sizes of FITC-conjugated dextran with 6 ROIs being selected for diffusivity analysis. (**F**) Time-lapse fluorescence images displayed with fire intensity look up table of dual parallel channel CHIPS undergoing VAPOR perfusion with FITC-conjugated dextrans ranging from 3 to 70 kDa. (**G**) ROI-based analysis of dextran fluorescence intensity over time beginning proximal to the dextran source channel (i) and ending distal to the PBS source channel (vi). (**H**) A schematic demonstrating increased pressure (+5 mmHg) within the dextran source channel driving diffusion into the perfusate from the PBS circulation that was sampled for spectrophotometric analysis. (**I**) Analysis of 40-kDa FITC-conjugated dextran fluorescence intensity within the PBS circulation after 24 hours of either normal (NP), or increased pressure (+5 mmHg, +10 mmHg) within the dextran source channel (means ± SD *P < 0.05 for *N* = 3 samples each, one-way analysis of variance (ANOVA) with Tukey’s post-test). (**J** and **K**) Time-lapse images and line profile analysis of fluorescence intensity at 0, 6, and 12 hours calculated as a ratio of the HP image divided by the NP image to reveal pressure-dependent regions of increased diffusion. hr., hours.

To further assess molecular diffusion within an acellular CHIPS, a dual parallel channel design separated by a thin 500-μm collagen wall (Fig. 4A) was designed for efficient real-time fluorescence-based analysis. The dual-channel CHIPS were FRESH printed, verified by OCT (Fig. 4B), and then perfused within the VAPOR bioreactor (Fig. 4C). Laminar flow (*Re* = 0.55) and channel integrity were confirmed by perfusion and tracking of fluorescent microbeads within the channels (Fig. 4D and movie S4). We observed laminar flow particle profiles, with a mean speed of 0.91 ± 0.30 mm/s (theoretical value calculated as 1.2 mm/s for the center of the channel). Next, we mimicked diffusion of biomolecules of different molecular weights using fluorescein isothiocyanate (FITC)–conjugated fluorescent dextrans from 3 to 70 kDa in one channel as a source with the opposing channel perfused with 1× phosphate-buffered saline (PBS) as a sink (Fig. 4E). Time-lapse fluorescence imaging of defined ROIs expanding outward from the dextran-perfused channel (Fig. 4F and fig. S3, A and B) established that diffusion throughout the CHIPS was molecular weight-dependent (Fig. 4G and movie S4). The 3-kDa dextran reached steady-state diffusion at a distance 1 mm (ROI 1) from the source channel within 3 hours, while the 70-kDa dextran reached a steady-state rate at ~20 hours. The diffusivity of larger 40- and 70-kDa molecules is notable compared to cast solid hydrogel scaffolds where diffusion has been reported to be much lower (*27*). To increase molecular diffusion and mimic post-capillary constriction, we introduced a 5- or 10-mmHg pressure differential (*28*) between the dextran source and PBS sink channels (Fig. 4H). Spectrophotometric analysis of the PBS sink perfusate after 24 hours showed significantly more 40-kDa dextran present in the systemic PBS circulation following a pressure increase of either 5 or 10 mmHg (Fig. 4I). Comparing the ratio of fluorescence intensity between high pressure (HP, + 10 mmHg) and normal pressure (NP) over time showed that increasing pressure drove the dextran into the PBS channel as well as throughout the CHIPS and into the furthest distal regions (Fig. 4, J and K; fig. S3C; and movie S4). Together, these results establish that molecules with a wide range of molecular weights can diffuse from the CHIPS channels and into the bulk of the scaffold, something that is not possible with the vast majority of microfluidic and organ-on-a-chip devices made from PDMS or plastics.

### Spatially patterned ECM and growth factors via multi-material FRESH printing

**Fig. 5.**
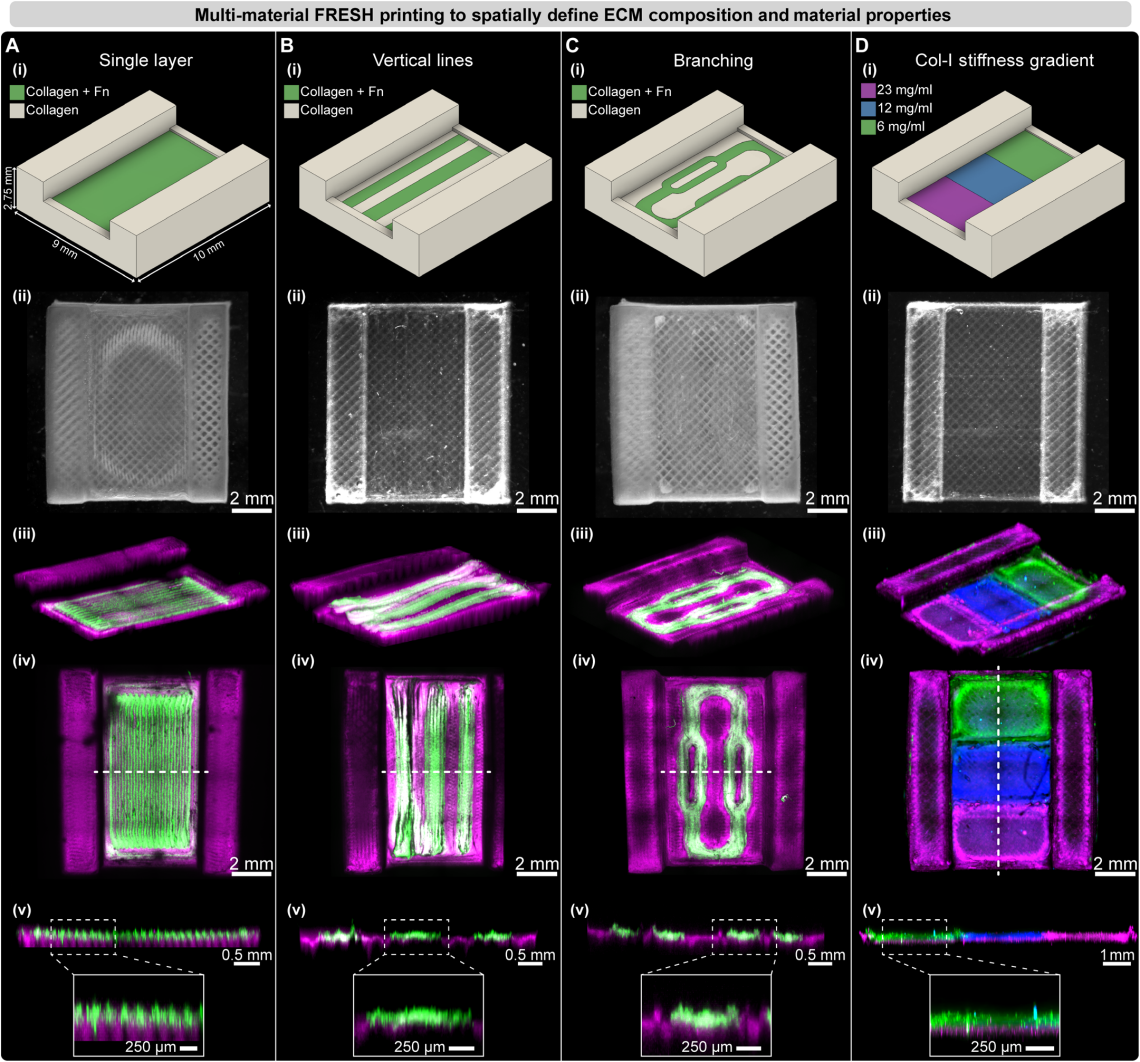
Multi-material FRESH printing to volumetrically define ECM composition, material properties, and growth factor localization. (**A** to **C**) A parallel plate style CHIPS design with a collagen frame and uniform single layer (A), vertical lines (B), and branching network (C) of fluorescently tagged Fn-containing collagen displayed as (i) CAD design and stereomicroscopy image, and (ii) quantified for multi-material printing registration with 3D confocal microscopy [(iii to v), dotted line indicates cut plane; inset scale bar, (v) 250 μm]. (**D**) A collagen stiffness gradient generated by multi-material printing of 6, 12, and 23 mg/ml collagen in adjacent regions within the parallel plate CHIPS (i), stereomicroscopy image (ii) of the three-material print demonstrates high-fidelity and multi-material registration upon fluorescence image analysis (iii to v).

The spatial patterning of cells and ECM within internal regions of perfusable tissue scaffolds is one of the major challenges faced when engineering more sophisticated organ-on-a-chip systems. To address this, we first developed a high-performance multi-material 3D bioprinter with three Replistruder 5 syringe pump extruders for bioink deposition (fig. S4 and movie S5). Next, we built a dual-camera optical needle alignment system to ensure micrometer-precise XYZ needle positions for each extruder (movie S5). For print validation and multi-material demonstration, we designed a parallel plate–style CHIPS containing an open rectangular channel to facilitate direct imaging of the FRESH-printed and patterned biomaterials (Fig. 5). Within the channel of the CHIPS, we created three unique geometries to highlight the versatility and accuracy of our improved multi-material FRESH printing. Collagen bioinks doped with fluorescently labeled fibronectin (Fn) were patterned as (i) a uniform layer ~200 μm in thickness across the entire channel (Fig. 5A and movie S5), (ii) vertical lines 1 mm wide by 145 μm in thickness running the length of the channel (Fig. 5B), and (iii) a defined branching pattern 145 μm in thickness with segments down to 500 μm in width within the main channel (Fig. 5C and movie S5). In addition, to demonstrate our ability to pattern ECM of varying stiffness within the same CHIPS, we printed softer and stiffer collagen regions (6, 12, and 23 mg/ml) along the length of the channel each labeled with different fluorescent dyes (Fig. 5D). Together, these results establish our ability to precisely register multiple extruders, spatially localize up to three biomaterials, and to pattern varied ECM material properties within CHIPS while maintaining micrometer-level registration.

Following validation and optimization of our multi-material printing platform, we sought to implement multi-material FRESH printing within our dual-channel CHIPS to pattern cells and ECM in 3D. Within our dual-channel design, our goal was to print a thin layer of collagen (12 mg/ml) + fluorescently labeled Fn along the channel lumen to encourage endothelial cell attachment to an Fn-containing substrate. In addition, we chose to pattern collagen (23 mg/ml) + vascular endothelial growth factor (VEGF) with a different fluorescently labeled Fn for visualization within the central region between the two channels to demonstrate localized growth factor deposition (fig. S5A). The dual-channel multi-material CHIPS was FRESH printed, optically cleared, and imaged via confocal fluorescence microscopy for visualization of the internally patterned structures (fig. S5B and movie S6). For both the collagen (12 mg/ml) channel linings and VEGF-containing central region, we observed accurate 3D spatial patterning (fig. S5C and movie S6). Multi-material dual-channel CHIPS also displayed high print fidelity with internal features closely matching expected values, demonstrating the multi-material printing system maintains print precision (fig. S5, E to I).

To test cell adhesion and growth within the multi-material patterned dual-channel CHIPS, human umbilical vein endothelial cells (HUVECs) were perfusion seeded at a high cell density (fig. S5D). Following 5 days of static culture, the HUVECs then spread along the channel lumen, formed a visible monolayer, and stained positive for the endothelial marker CD-31 (fig. S5, J to N, and movie S6). While there was some evidence of cell migration into the bulk scaffold, the HUVECs were primarily localized to the channel lumen into which they were seeded. We next wanted to determine the influence of shear stress on cell adhesion for perfusion seeded endothelial cells within CHIPS. VAPOR was modified for perfusion of a parallel plate design to image cell monolayer formation and adhesion under flow without requiring tissue clearing (fig. S6, A to C). During static culture, HUVEC endothelial cells formed confluent endothelial monolayers on parallel plate CHIPS and displayed robust CD-31 expression (fig. S6, D and E). However, under modest shear stress (0.1 dyne/cm^2^), surface seeded endothelial cells detached over time (fig. S6, F to E). This is why we evaluated whether using multi-material printing it would be possible to more robustly pattern endothelial cells within CHIPS.

### FRESH-printed multicellular CHIPS

Whether for in vitro systems or future applications implanting in vivo, solely relying on luminal perfusion seeding cannot achieve cell densities required for most tissue types. Therefore, to increase cellularization within CHIPS, we implemented multi-material printing of cell-laden bioinks. The process for printing cellular bioinks with FRESH is similar to that of collagen, except that rather than using a pH change to initiate the gelation of a collagen bioink, the cells are incorporated into a fibrinogen bioink and thrombin is used to enzymatically trigger the cross-linking of fibrinogen into fibrin. Since both collagen and cell-laden fibrinogen bioinks have orthogonal gelation mechanisms, we can FRESH print both biomaterials into the same support bath to create a fully integrated, multi-material, cellularized CHIPS.

**Fig. 6.**
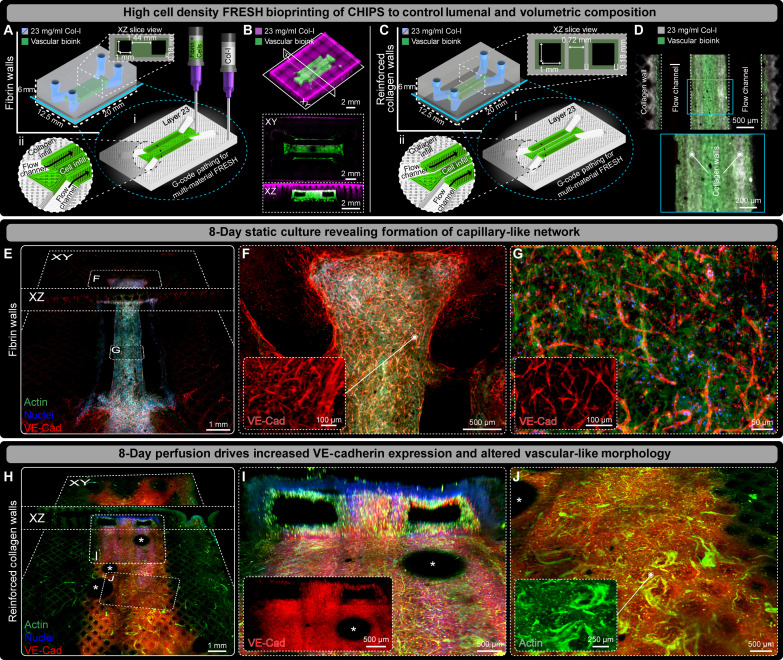
Multi-material printing of vascular cell bioink drives capillary-like network formation and enhanced VE-Cad expressing within VAPOR perfused CHIPS. (**A**) Schematic and 3D printer machine pathing (G-code) of dual parallel channel multi-material CHIPS with an internal fibrin-based vascular cell bioink (HUVEC and MSC) region containing fluidic channels. (**B**) 3D confocal fluorescence imaging of optically cleared high-fidelity collagen (magenta) and vascular cellular bioink (green) printing with XY and XZ plane views revealing internal structure, open channels, and volumetric registration between the collagen and vascular bioink. (**C**) Schematic design and G-code of dual-channel CHIPS with collagen-reinforced walls between the cellular channels. (**D**) XY mid-plane view of whole-mount confocal fluorescence images of optically cleared 1-day statically cultured vascular CHIPS with collagen walls. Combined multiphoton fluorescence and second harmonic imaging of the vascular bioink and collagen shows spatial alignment between the cellular biomaterial (green) and collagen walls (gray). (**E**) Both XY and XZ midplane slice views from 3D confocal imaging of optically cleared dual parallel channel cellular CHIPS with fibrin walls statically cultured for 8 days and stained for actin (green), nuclei (blue), and the endothelial marker VE-Cad (red). (**F** and **G**) Confocal images of regions within (E) revealing microvascular cell spreading and migration (F) and vascular-like network formation (G) with inset displaying magnified views of VE-Cad only channel. (**H**) Both XY and XZ slice views of the channel bottom surface from 3D confocal imaging of optically cleared dual parallel channel cellular CHIPS with collagen walls following 8 days of perfusion culture within VAPOR. (**I** and **J**) Confocal images revealing strong luminal expression of VE-Cad (I) and evidence of cellular remodeling and vascular-like network maturation (J) with inset images displaying magnified views of VE-Cad and Actin only channels. *Indicates air bubble artifacts introduced during CHIPS optical clearing and imaging.

To encourage formation of capillary-like networks within our CHIPS, we created a fibrinogen-based vascular bioink containing HUVECs and human bone marrow–derived mesenchymal stem cells (MSCs) to support endothelial cell function (*29*). We evaluated multiple cellularized CHIPS designs to identify how cellularization affects CHIPS stability and structure over time. First, our vascular bioink (30 million cells/ml 90% HUVECs + 10% MSCs + fibrin) was printed as both the channel lining and dividing region between dual parallel fluidic channels, while the remaining CHIPS structure was collagen (Fig. 6A). Note that all subsequent cell-laden bioinks did not contain VEGF as we wanted to evaluate cell behavior in the absence of exogenous growth factors. Confocal imaging of the CHIPS immediately after printing confirmed our ability to volumetrically pattern both the cellular and collagen bioinks at high precision in 3D while maintaining patent fluidic channels (Fig. 6B and movie S7). However, after static culture for 8 days, this CHIPS design resulted in buckling along the midline likely due to cell-driven compaction forces (fig. S7A). To prevent buckling and maintain proper perfusion in the VAPOR bioreactor, we added internal collagen walls between the channel linings and central region as mechanical reinforcement (Fig. 6C). Multiphoton and second harmonic generation imaging of the collagen-reinforced CHIPS revealed high-fidelity cellular channel linings and the presence of the extra collagen walls between the fibrin-based vascular channel lining and the central cellular region (Fig. 6D). After 8 days of static culture, the mechanical reinforcement provided by the collagen walls was sufficient to prevent the CHIPS from buckling compared to the fibrin-wall only CHIPS (fig. S7B). These results highlight the importance of both CAD design and material properties for long-term success and performance of cellularized CHIPS and our ability to rapidly make such changes.

Next, we wanted to evaluate the influence of the added collagen walls to promote vascular endothelial cadherin (VE-Cad) expression and an elongated cell morphology during extended periods of culture. After 8 days of static culture, the CHIPS with internal fibrin walls exhibited large areas expressing VE-Cad (Fig. 6E). Despite the absence of perfusion, VE-Cad^+^ capillary-like structures were observed within and adjacent to the cellular printed regions (Fig. 6, E to G, and movie S7). Specifically, we observed VE-Cad^+^ networks spanning distances >2 mm, cellular migration from the ends of the printed cellular regions into the acellular collagen, and dense VE-Cad^+^ capillary-like networks throughout the central dividing region (Fig. 6, E to G, and movie S7). In CHIPS with collagen-reinforced walls, we observed similar results after 8 days of static culture (fig. S8A and movie S7), with VE-Cad expression along the channel lumen (fig. S8B), and migration of cells (>1 mm) beyond the edge of the printed cellular regions from where the cells originated (fig. S8C). The addition of the collagen reinforcement did not appear to deter endothelial cell migration or VE-Cad expression. Moreover, these results are clear evidence that cells can preferentially migrate along printed collagen filaments within the infill of our CHIPS model.

While static culture conditions were sufficient to induce VE-Cad^+^ expression, the addition of shear stress via perfusion has been shown to increase endothelial cell–specific protein expression and promote a vascular-like morphology (*30*). To test the effects of perfusion in our system, we performed 8 days of VAPOR perfusion culture for each CHIPS design. In CHIPS with fibrin walls (fig. S8D), we observed high levels of VE-Cad expression encircling the printed channels (fig. S8E and movie S7) and cell elongation parallel to the direction of flow within the central dividing region (fig. S8F and movie S7). Similarly, CHIPS with collagen walls after 8-days of perfusion exhibited extensive cell migration throughout the scaffold and formation of VE-Cad^+^ vascular-like structures within the bulk scaffold. These structures were detected far from where the HUVECs and MSCs were initially printed (Fig. 6H). In addition, the vascular CHIPS with collagen walls did not deform during perfusion in VAPOR resulting in increased channel patency compared to the fibrin only wall CHIPS (Fig. 6I and fig. S8D). Lastly, we observed evidence of a perfusion-stimulated vascular-like morphology reminiscent of larger diameter microvessels within the VE-Cad^+^ zones around the fluidic channels (Fig. 6J and movie S7). These results demonstrate that the addition of collagen walls helps to reinforce CHIPS, encourages VE-Cad^+^ cell elongation, and prevents scaffold buckling to ensure sustained VAPOR perfusion of vascular CHIPS. Further, the increased shear stress resulting from perfusion promotes cellular assembly into VE-Cad^+^ vascular-like networks spanning 8 to 100 μm in diameter throughout the scaffold in only 8 days’ time.

### Pancreatic-like CHIPS demonstrate glucose-stimulated insulin response

We next sought to engineer a cellularized CHIPS system that can secrete proteins and demonstrate the structural complexity and more advanced function that can be achieved. Specifically, we focused on glucose-stimulated insulin secretion (GSIS) performed by islets of the pancreas, the failure of which leads to type I diabetes. The engineering of pancreatic tissue has made several advances, including hydrogel-based encapsulation to evade the immune system (*31**,* *32*) and organ-on-a-chip systems containing isolated islets (*33*–*35*), showing that implantation of insulin-producing engineered tissues has therapeutic potential (*33*–*36*). However, there has yet to be a perfusable tissue construct created entirely out of native ECM and cells with the potential for future implantation.

To model the GSIS of pancreatic tissue, mouse MIN6 cells were chosen as they exhibit beta cell–like secretion of insulin in response to glucose. MIN6 cells were incorporated into our vascular bioink to form a high-concentration (60 million cells/ml total 50% MIN6 + 45% HUVEC + 5% MSC) fibrin-based pancreatic-like bioink. This pancreatic-like bioink was multi-material FRESH printed into the dual-channel CHIPS design and confirmed to maintain geometric stability during 8 days of static culture using OCT image analysis (fig. S9). Our next goal was to confirm that both the HUVECs and MIN6 cells expressed the appropriate vascular and islet markers when combined and printed as a single cellular bioink. The pancreatic-like CHIPS were FRESH printed and statically cultured for 8 days to allow sufficient time for vascular-like network formation and insulin expression (fig. S10, A to C). After fixing and imaging the CHIPS, we observed dense cellularization in the cell printed regions as well as cell migration into the surrounding collagen scaffold (fig. S10D). In addition, the internal CHIPS structure was maintained throughout the culture period as evidenced by the patent fluidic channels (fig. S10E). MIN6 cells stained positive for insulin within both the central dividing region and adjacent channel walls (fig. S10, E to G). In addition, within the printed cellularized regions, CD-31^+^ staining marked the formation of capillary-like networks (fig. S10H), and cell migration was guided by the printed collagen filament angle (ex. 45° on the surface) (fig. S10, I and J). These results together suggest that the topology and direction of printed filaments within the CHIPS acellular volume can serve as a potential pathway to define cell migration, network density, and possibly vascular-like branching. Further, we show that a combined HUVEC-MSC-MIN6 pancreatic-like bioink can both encourage vascular-like expression of CD-31 and pancreatic-like expression of insulin.

We also evaluated CHIPS physical stability given the increased cell concentration in these devices. At distances 200 μm into the collagen scaffold and away from the cellular regions, where the filament pattern forms a cross-hatched infill pattern, cell migration followed the filament direction (fig. S10, K and L). When examining internal deformation as evidenced by changes in the infill structure, we observed minimal changes in the infill-infill lattice spacing (378 ± 30 μm compared to 391 μm theoretical). We did observe a small increase in the filament width (116 ± 18 μm compared to 106 μm before culture), which is likely the result of either cell migration covering the collagen filaments and/or slight swelling over time. In addition, the infill void space distance decreased from 301 μm to 269 ± 20, suggesting that the internal structure of the CHIPS is stable for 8 days. Thus, for this specific CHIPS design, deformation is at most ~10% depending on the metric measured, most notably being decreased infill porosity as the cells migrate and cover the initially acellular collagen.

**Fig. 7.**
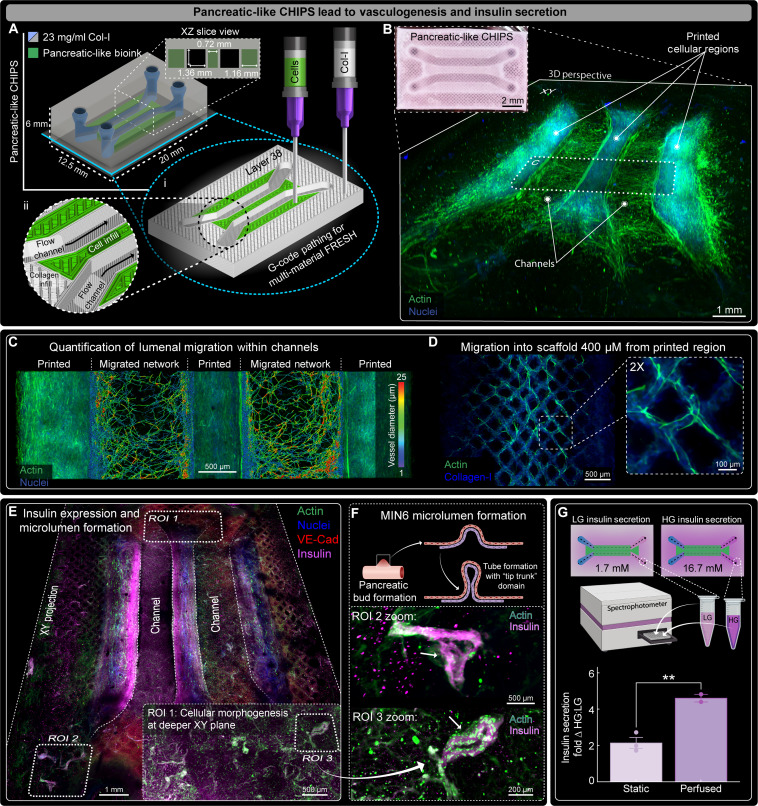
FRESH-printed vascular pancreatic CHIPS demonstrate vasculogenesis and insulin secretion. (**A**) Schematic design and 3D printer machine pathing G-code of a dual parallel channel multi-material CHIPS with pancreatic vascular cell bioink (MIN6, HUVEC, and MSC) regions surrounding both sides of the channels. (**B**) Pancreatic CHIPS FRESH printed and visualized via bright-field stereomicroscope (inset) and 3D confocal fluorescence imaging of the optically cleared pancreatic scaffold after 12 days of static culture. (**C**) XY midplane view from confocal fluorescence image of the 12-day statically cultured pancreatic CHIPS following 3D vascular network segmentation for quantification of network diameter and density within the migratory zones. (**D**) Example confocal fluorescence images revealing additional cell migration into the acellular regions of the CHIPS beneath the cellular regions guided by the printed collagen filaments. (**E**) XY midplane projection view from 3D confocal fluorescence imaging of the optically cleared 12-day VAPOR perfused pancreatic CHIPS. (**F**) Graphic illustration and example ROIs depicting evidence of early MIN6 pancreatic bud and microlumen formation with actin (green) and insulin (magenta) fluorescence images from ROIs 2 and 3 in (E). (**G**) Graphic illustration and quantification for insulin secretion ELISA assay from 1.5-hour glucose-stimulated [ratio of high glucose (HG) to low glucose (LG)] insulin secretion experiment between 12-day static and perfusion cultured pancreatic CHIPS (means ± SD; **P < 0.01 for *N* = 3 static tissues; *N* = 2 perfused tissues, unpaired *t* test).

While the static pancreatic-like CHIPS did exhibit insulin expression, a large volume of our construct remained acellular. In principle, since our entire scaffold is fabricated from ECM, we can increase the total cellular volume within the CHIPS by printing additional cellularized regions surrounding our perfusable channels. Therefore, a modified pancreatic-like CHIPS was designed with three distinct cellularized regions between and adjacent to each side of the perfusable channels to increase the printed cell volume by 50%. In addition, to strengthen the device and prevent cell-mediated deformation during VAPOR perfusion, we included collagen walls surrounding the channel linings similar to our vascular-like CHIPS (Fig. 7A). The CHIPS were successfully FRESH printed and after static culture for 12 days, displayed extensive cell migration into adjacent acellular regions (Fig. 7B and movie S8). Notably, the initially acellular collagen channel lining now had a cellularized lumen with visible vascular-like networks that connected the printed regions surrounding the perfusable channels (Fig. 7C, fig. S11A, and movie S8). In addition to the cell migration into and around the fluidic channels, extensive migration was observed outward into the surrounding collagen scaffold up to a full millimeter from the initial printed regions (fig. S11, B and C). Again, the channel lumens remained patent, and the originally acellular walls were now densely populated with cells (fig. S11, D to G). Quantification of the branching endothelial cells network formed between the channels revealed a mean diameter of 9.3 ± 2.7 μm (Fig. 7C and movie S8). Because of the extended 12-day culture, cell migration was detected hundreds of micrometers into the CHIPS infill space where they continued to migrate along the printed collagen filaments (Fig. 7D and movie S8).

Next, we perfused the pancreatic-like CHIPS in the VAPOR bioreactor for 12 days to improve nutrient delivery, increase cell proliferation and growth, and enhance MIN6 insulin secretion. Perfusion increased the extent of cellularization and revealed cell migration from printed cellular regions inward to coat the channel lumen and outward along collagen filaments (Fig. 7E, fig. S12, and movie S9). Both initially acellular, channel lumens now displayed a VE-Cad^+^ cell layer (fig. S12, A to C), while we observed de novo VE-Cad^+^ single vascular-like bundles and Y-branching networks ranging in diameter from 25 to 100 μm in the infill void space (fig. S12, D to G, and movie S9). Within all printed cellular regions, perfusion increased insulin expression, and insulin^+^ structures were detected far from the printed cellular regions (Fig. 7E and movie S10). One of the most exciting observations was the presence of looping structures that contained insulin^+^ MIN6 cells and actin^+^ HUVECs and/or MSCs, reminiscent of early pancreatic morphogenesis observed during islet development (Fig. 7F and movie S10) (*37*–*39*).

Physical dimensions after perfusion were different than static culture, with channel width and height significantly increasing (fig. S13). In both static and perfused conditions, the channel height was smaller than the channel width (fig. S13B) likely due to dehydration during tissue clearing and the compression of the channels to prevent drift during image tile scanning. Channel perimeter also increased in the perfused CHIPS, suggesting that the positive pressure accompanying fluid flow resists the cellular compaction forces to maintain open and patent channels (fig. S13C). Similarly, the inlet and outlet spacing of the perfused pancreatic-like CHIPS closely matched the original CAD design and was significantly larger than the static CHIPS (fig. S13, D to H).

To confirm that the MIN6 insulin^+^ staining was indicative of functionally secreted insulin in response to glucose stimulation, we performed a GSIS assay coupled with an insulin enzyme-linked immunosorbent assay (ELISA) comparing the static and perfusion-cultured pancreatic-like CHIPS. GSIS results showed that pancreatic-like CHIPS cultured statically produced a stimulation index (SI) of 2, whereas those cultured under perfusion exhibited a significantly higher SI of 4.6 (Fig. 7G). This places the performance of pancreatic-like CHIPS beyond previous reports for MIN6 pseudo-islet spheroids and approaching primary pancreatic islets (SI ~10) (*33*–*35*, *40*–*43*). Last, we quantified the amount of constitutive insulin secreted into the perfused media over 24 hours and detected >8 ng of insulin produced by the pancreatic-like CHIPS. Together, these results demonstrate that bioprinting beta cell–like MIN6 in a fully biologic perfusable 3D ECM environment with endothelial and stromal cells drives the formation of an engineered tissue construct with both responsive and constitutive secretory function. While these initial studies show translational potential for the pancreatic-like CHIPS, further functional improvement is likely achievable through multiple approaches including increasing the beta-like cell volume within the construct, extending the culture time to promote cell maturation, and incorporating fully differentiated human iPSC-derived beta-like cell clusters or human primary islets.

## DISCUSSION

In this work, we have developed an advanced scaffold biofabrication platform centered on FRESH 3D bioprinting of CHIPS, achieving microfluidic network designs with superior resolution and print fidelity compared to any other extrusion-based bioprinting approach. By using CAD to easily iterate scaffold design, we enable rapid iteration and reproducible multi-material biofabrication with over three distinct bioinks. This CHIPS methodology allows for the precise engineering of complex tissue structures, pushing the boundaries of what is possible in terms of biofabrication. This is all combined with our customizable perfusion bioreactor system, VAPOR, featuring integrated barbed fittings that seamlessly interface with the collagen-based CHIPS, enabling long-term perfusion. This system ensures that the engineered tissues receive the necessary nutrients and oxygen, mimicking the natural physiological environment more closely. Additional advances include an alignment system that facilitates precise local spatial patterning of multiple ECM, cell, and protein-laden bioinks within CHIPS, achieving a resolution of ±20 μm within centimeter-scale scaffolds, and a direct printing approach that eliminates the laborious process of perfusion seeding scaffolds post-3D printing. Overall, this technology provides previously undiscovered capabilities for organ-on-a-chip systems, disease modeling, and the creation of functional tissue constructs.

The combination of CHIPS and VAPOR has both advantages and limitations when compared to established microfluidics. Microfluidics fabricated using photolithography excel at producing smaller features on the order of 10 μm that remain challenging to replicate using extrusion-based bioprinting. In our current system, the lower limit for creating perfusable channels is ~100 μm in diameter. However, this is not constrained by the precision of our 3D printer (200-nm resolution) or the needle size (as small as 20-μm diameter) (*19*) but rather by the average size of gelatin microparticles within the support bath. Presently, the average microparticle size is ~25 μm, necessitating that the minimum perfusable channel diameter be several particles wide to account for the random packing arrangement in the bath and filament diffusion before gelation. The coacervation process used to make the support bath can be fine-tuned to produce smaller ~10-μm microparticles. While this provides a pathway toward enhanced resolution in future studies, we expect that ~50-μm-diameter channels are the lower limit for CHIPS even with these improvements. Fortunately, endothelial cells and pericytes can self-assemble into smaller capillary-scale vessels that may obviate the need for higher resolution in CHIPS. Cellularized CHIPS exhibit extensive cell migration along printed collagen filaments throughout, self-assembling into vascular-like networks composed of VE-Cad^+^ and CD-31^+^ cells within regions that would typically be inaccessible in conventional microfluidic devices. CHIPS also enable patterning of multiple cells and materials into complex 3D architectures within fully enclosed compartments not possible with perfusion seeding after microfluidic device fabrication.

Whether perfusion seeding or direct printing of HUVECs is better remains an open question and is likely context dependent. Perfusion seeding of endothelial cells has been the de facto standard in microfluidics (*44*, *45*), 3D bioprinting (*46*, *47*), and decellularization/recellularization (*48*, *49*) because until now, there have been few other viable options. While this works at a basic level, one the problems that continue to challenge perfusion seeding is achieving uniform endothelial cell coverage over the entire luminal surface (*50*). Our results show that perfusion seeding also works in CHIPS to produce endothelial cell monolayers under static conditions, but the cells delaminate under flow even at low shear stress of ~0.1 dyne/cm^2^. It may be possible to solve this issue through modifying the collagen bioink to improve cell adhesion, but we attempted a different approach. Instead, we used direct cellularization by FRESH 3D bioprinting HUVECs within a fibrin bioink along the channel lumens. While we did not observe formation of endothelial monolayers equivalent to static seeding, we were still able to pattern viable cells that went on to migrate throughout the CHIPS and form capillary-like networks spanning from the printed channels into the bulk scaffold, a unique result.

One potential limitation of this direct cellularization approach compared to traditional perfusion seeding is the extended duration of culture required to achieve a confluent endothelium-like surface. Since the HUVECs are embedded within a fibrin biomaterial and must migrate into the channel lumen, additional time is necessary for this process. It is currently believed that tissues prevascularized through static culture produce naïve, unrefined vascular networks that, upon anastomosis with host vasculature, result in slow meandering blood flow that can clot within the prevascularized network (*51*, *52*). As flow is a crucial source of information for vascular network formation and remodeling, it is plausible that perfusion-based culture can refine prevascularized networks. If instead, endothelial monolayer formation is the goal, precoating the CHIPS channels with Fn or additional ECM may help overcome the observed detachment following applied shear stress. Overall, direct cellularization of channel lumens build upon perfusion seeding by providing an additional approach to build endothelialized channels.

With any new technology, there can be questions over ease of use and adoption due to the need for custom equipment, technical know-how, and overall affordability; however, we do not foresee this being a barrier for CHIPS and VAPOR. Specifically, FRESH 3D bioprinting has an established track record of widespread adoption that has been facilitated by publishing a range of open-source 3D bioprinter hardware designs and offering annual training workshops. Here, we used a custom built three-material bioprinter based on a commercial Aerotech motion control platform that achieves 1-μm accuracy, similar to systems in multiple academic laboratories (*53*–*56*). Although this hardware produces excellent results, it is not required for high-fidelity FRESH printing. To create CHIPS reliably, a user requires a 3D printer capable of 20-μm accuracy or better. This performance threshold is on par with the reported specifications of most commercial 3D bioprinters, such as the CellInk Bio X6 with a reported 1-μm accuracy, which offers official support for FRESH printing. This performance can also be achieved via our published guides to convert affordable (e.g., <$1500) desktop plastic 3D printers such as the FlashForge Finder with 11-μm reported accuracy into 3D bioprinters using open-source syringe pump extruders and Duet3D electronics (*19*, *57*).

Here, we expand upon this work for multi-material printing with our open-source optical needle alignment system capable of ~3 μm accuracy in the *x*, *y*, and *z* axes. With precisely registered needle tips, the fidelity of our single-material printing extends to every additional bioink used in multi-material printing. While needle alignment adds an additional setup, our current system consistently produces prints <20-μm error. Furthermore, to facilitate the adoption of our technology and reduce the barrier to entry, more than 50 laboratories have been trained in FRESH in 5 years through in-person open-source 3D bioprinting workshops hosted at Carnegie Mellon University (*58*, *59*).

Similarly, the VAPOR bioreactors are fabricated using widely available commercial stereolithography apparatus (SLA) 3D printers such as the Form3B and off-the-shelf biocompatible photoresins. This, and the widespread transition from STL (meshed part files) to 3MF (3D manufacturing format) file formats vastly improves the dissemination potential of FRESH, as 3MF files embed both the 3D CAD model and all software slicing settings within a single file. This file can be opened by another user and replicated or edited as necessary. Compared to STL, 3MF files facilitate global sharing between laboratory group researchers and enable advanced capability for users currently familiar with FRESH without the need for independent optimization.

Looking forward, the development of fully biologic scaffolds is driven by our long-term goal to engineer functional volumetric tissue for therapeutic applications. Standard materials used in microfluidics such as PDMS, plastics, and photoresins, while potentially implantable, lack the ability for extensive host cell infiltration, remodeling, and in vivo integration. Indeed, these biomaterials are widely known to elicit fibrotic encapsulation due to the foreign body response (*60*). In contrast, acellular FRESH-printed collagen scaffolds have been implanted in vivo and demonstrated the ability to support extensive cell infiltration and host-mediated vascularization without any evidence of fibrosis (*19*, *61*). This is the same bioink as used in the CHIPS, and thus the biological response should be comparable as long as the cells we are integrating are compatible with the host.

While microfluidic-inspired designs serve as a starting point for our CHIPS in the current work, we acknowledge that CHIPS will require design modifications for application-specific in vivo implantation. Notably, our results indicate that in vitro VAPOR perfusion promotes lumen patency compared to static cultured controls, potentially resisting cellular compaction. Consequently, it is plausible that if the perfusable fluidic networks can be directly anastomosed to the host vascular system, blood flow and pressure will maintain vessel patency. In addition, the collagen concentration in CHIPS can be tuned over a broad range, and other collagen isoforms including fibrillar collagen types II and III can also be incorporated to tailor scaffold stability (*62*). In vivo stability will likely also require altering the inlet and outlet termination of CHIPS to incorporate a high-concentration collagen sewing cuff-like region, structurally reinforcing acellular areas of CHIPS with higher-concentration collagen and/or incorporating structural reinforcing fibers (*63*) to serve as load-bearing regions to minimize in vivo compression. Last, FRESH printing has demonstrated the ability to print fibrin-based cell-laden bioinks at concentrations of 100 to 500 million cells/ml (*19*, *64*, *65*), higher than any other bioprinting approach. Thus, the cell density within CHIPS can be tailored to achieve greater cell mass within a smaller footprint, thereby maximizing the achievable therapeutic dosage for the patient. As a potential example, the pancreatic-like CHIPS while developed here as an in vitro application, could be adapted to study cellular remodeling and long-term in vivo stability and function after VAPOR perfusion and subsequent host implantation.

In conclusion, the CHIPS and VAPOR platform represents a substantial advancement in 3D bioprinting technology, merging high-resolution FRESH printing with dynamic perfusion systems to engineer complex, functional, centimeter-scale tissue constructs. This approach pushes the boundaries of what is achievable in tissue engineering by enabling precise 3D multi-material biofabrication, direct compartment cellularization, capillary-like network formation, and the emergence of tissue-scale secretory function. While challenges remain—such as optimizing microchannel resolution, enhancing endothelial stability under flow, and refining in vivo integration—the flexibility and accessibility of this system pave the way for broader adoption and future innovation.

## MATERIALS AND METHODS

### 3D bioprinter setup

All FRESH printing was executed on custom-built 3D bioprinters using our open-source Replistruder 5 syringe pumps (*57*, *64*, *66*) (parts files available upon publication for download under a CC-BY-SA license found at https://3d.nih.gov/users/awfeinberg). Single-material printing used a custom gantry 3D printer configuration driven by four Parker Hannifin 404 × R 100 mm travel precision stages (8-μm travel accuracy) as previously described (*64*). For multi-material cellular bioprinting, a high-performance motion control system was designed using the AGS1000 platform (Aerotech Inc.). Three Replistruder 5 syringe pumps and a dovetail mounted OCT scanhead (Thorlabs) were each mounted to TU-30 linear ball screw stages (IKO) for independent actuation of each extruder. The custom bioprinter platform resulted in a build volume of 100 mm (X), 300 mm (Y), 100 mm (Z), and 80 mm for each TU-30 Replistruder 5 substage. A 500-μl, 1-ml, or 2.5-ml syringe (Hamilton) containing bioink with a stainless-steel needle of either 90-μm ID for collagen (Jensen Global, JG34-0.25HPX) or 150-μm ID for cellular bioinks (Jensen Global, JG30-0.5HPX) was used.

### Collagen bioink preparation

Unless stated otherwise, an acidified collagen bioink (23 mg/ml) was used for all prints and prepared as previously described (*19*). Briefly, sterile neutral collagen bioink (35 mg/ml; LifeInk 200, Advanced Biomatrix, 5278) was diluted in a 2:1 volume ratio with 0.24 M acetic acid (VWR, 97064-482) or sterile acidified collagen bioink (35 mg/ml; LifeInk 240, Advanced Biomatrix, 5267) was diluted in a 2:1 volume ratio with sterile deionized (DI) H_2_O and mixed back and forth 40 times between two mated syringes. For the preparation of fluorescent bioinks, 1 ml of acidified collagen bioinks were mixed with 10 to 20 μl of human Fn (500 μg/ml; Corning, 356009) fluorescently conjugated to either Alexa Fluor 405, 488, 555, and 633 *N*-hydroxysuccinimide esters (*67*, *68*). In multi-material printing experiments, the final concentration of the Fn within the collagen bioink was 50 μg/ml. Some fluorescently tagged bioinks also contained VEGF [recombinant human VEGF-165 (PEPROTECH, 100 to 20) at 50 ng/ml]. In all cases, syringes containing acellular bioink were centrifuged at 3000*g* for 5 min at room temperature to remove any air bubbles generated during the preparation process. The bioink was then transferred to a Hamilton glass syringe for printing.

### Cell culture

Unless otherwise stated, cells were cultured at 37°C under 5% CO_2_ with media supplemented with 1% (v/v) penicillin-streptomycin being exchanged every 2 days. Pooled HUVECs (Lonza, CC-2519) were cultured in endothelial cell media (Lonza, EGM-2, CC-3162). Bone marrow–derived MSCs [American Type Culture Collection (ATCC), PCS-500-012] were cultured on flasks coated with quick-coating solution with growth media (ATCC, PCS-500-041). Adult human dermal microvascular endothelial cells (HDMECs) (Lonza, CC-2543) were cultured on flasks coated with quick-coating solution in microvascular endothelial cell media (Lonza, EGM-2 MV). MIN6 (mouse insulinoma cell line) (Addexbio, C0018008) were cultured in high-glucose Dulbecco’s modified Eagle’s medium (DMEM, Gibco, 11-965-092) with 15% fetal bovine serum (v/v), 2 mM sodium pyruvate, 20 mM Hepes, and 0.05 mM β-mercaptoethanol, using passages 9 to 15. MIN6 cells were seeded at a density of 2 × 10^4^ cells/cm*^2^* and passaged when they reached 80 to 90% confluency.

### Cellular bioink preparation

Vascular and pancreatic bioinks were prepared for cellular bioprinting experiments following similar protocols. For the vascular bioink, HUVECS (passage 4 to 6) and MSCs (passage 2 to 4) were cultured following the previously described procedure and lifted using a trypsin-EDTA solution. Trypsin was neutralized using trypsin-neutralizing solution with 7.5 μM bivalirudin (Cayman Chemical, 23035) as a thrombin inhibitor at a 1:2 ratio. The cells were pelleted at 200*g* for 5 min and then resuspended in 1 ml of Hanks’ balanced salt solution (HBSS, Gibco, 14175-095). MSCs (1 × 10^6^) and HUVECs (9 × 10^6^) were transferred into a 1-ml BD syringe. This syringe was then centrifuged at 190*g* for 5 min, and the supernatant was aspirated until approximately 100 μl remained. Fibrinogen (165 μl of 120 mg/ml; MilliporeSigma, 341573) and 65 μl of 5% xanthan gum (dissolved in HBSS) were loaded into a separate 500-μl Hamilton gastight syringe. The 1-ml and 500-μl syringes were connected with a female Luer lock adapter, and the fibrinogen, xanthan gum, and cells were mixed 50 times. The vascular bioink was centrifuged at 300*g* for 3 min in the 1-ml syringe to remove bubbles and transferred to the 500-μl syringe. The final vascular bioink consisted of 30 × 10^6^ cells/ml, fibrinogen (60 mg/ml), and 1.0% (w/v) xanthan gum. The pancreatic bioink was prepared similarly to the vascular bioink with the following adaptations. MIN6 cells (passage 9 to 15) were lifted using trypsin solution for 5 min, and 10 × 10^6^ MIN6 cells were added to the 1 × 10^6^ MSCs and 9 × 10^6^ HUVECs in a 1-ml syringe. The final pancreatic bioink consisted of 60 × 10^6^ cells/ml (30 × 10^6^ MIN6/ml, 27 × 10^6^ HUVEC/ml, 3 × 10^6^ MSC/ml), fibrinogen (60 mg/ml), and 1.0% (w/v) xanthan gum.

### FRESH support bath generation

Cellularized CHIPS were printed using a sterile support bath (LifeSupport, FluidForm) prepared according to the manufacturer’s instructions. When printing collagen-based bioinks, the support bath was rehydrated with a 2:1 mixture of cold 100 mM Hepes (pH 7.4) (Corning, 60-034-RO) and serum-free DMEM (Gibco, 11-965-092). When printing fibrinogen-based bioinks, the support bath was rehydrated with cold 100 mM Hepes (pH 7.4) and thrombin (1 U/ml; MilliporeSigma, T4648). Acellular CHIPS were printed using a FRESH support bath generated using a complex coacervation method as previously described (*19*). Briefly, FRESH v2.0 support bath (*19*) was made by dissolving 3.0% (w/v) gelatin type B (Thermo Fisher Scientific, G7-500), 0.3% (w/v) gum arabic (Sigma-Aldrich, G9752), and 0.125% (w/v) Pluronic F-127 (Sigma-Aldrich, P2443) in a 50% (v/v) ethanol solution at 45°C. Soon after dissolving, the pH of the solution was adjusted to 5.65 using 1 M hydrochloric acid. The solution was sealed and stirred overnight at room temperature. The following morning, the slurry was centrifuged at 300*g* for 2 min, the supernatant discarded, replaced with DI H_2_O, and shaken to wash the particles. The slurry was recompacted by centrifuging at 750*g* for 3 min, and the supernatant was discarded and replaced with DI H_2_O. This washing step was repeated for a total of three times. After the final round of washing, the slurry was resuspended to a final concentration of 100 mM Hepes (pH 7.4) and stored at 4°C. Before printing, the slurry was placed in a vacuum chamber at room temperature for 30 min followed by centrifugation at 2000*g* for 5 min. The supernatant was discarded, and the slurry was transferred into the print container of choice.

### CHIPS design

CHIPS were created in CAD software (Autodesk Inventor; Autodesk Fusion 360). All models were exported as STL files before printing. Overall design features and dimensions varied between experimental conditions and requirements. Perfusable networks were designed as void space within the printed CHIPS. All STL files used in the manuscript can be found at the Zenodo repository (10.5281/zenodo.14975240). The general pattern for perfusable CHIPS consisted of the following: overall size = 20 mm long–by–12.50 mm wide–by–6 mm in thickness; barbed cutouts are spaced 15.00 mm on center in length by 5.50 mm on center width; barb specifications consist of a shaft diameter = 1.75 mm, tip diameter = 1.60 mm, and a top of scaffold to barb tip distance = 2.273 mm; the overall perfusable network is centered on the CHIPS with 0.75 to 1 mm of collagen on the top and bottom. 3D renders for manuscript preparation were generated within the Fusion 360 rendering environment and exported as PNG files. Assembly videos of components were made within the Fusion 360 animation environment and exported as .avi or .mp4 files.

### FRESH 3D bioprinting of collagen type I

Collagen type I was FRESH printed as previously described (*19*). All STL files were sliced using slicing software (Ultimaker, Cura; PrusaResearch, PrusaSlic3r; Slic3r) to produce G-code files. For 80- and 150-μm ID needles, a layer height of 32 and 60 μm was used, respectively. Chips were printed at 23 to 70 mm/s with two perimeters, four top and bottom layers, and 35% infill. Retraction and combing were enabled to better control fluid flow and decrease print duration. G-code was optimized to avoid excessive skin overlap that causes rapid small printed segments movements. All constructs were printed at room temperature (22°C). Upon completion, the constructs were incubated at 37°C for at least 30 min to melt the support bath and release the print. The molten support bath was exchanged with warm print storage solution (PSS) consisting of 1× PBS, 50 mM Hepes (pH 7.4), and 1% (v/v) penicillin-streptomycin (Life Technologies, 15140-122). Acellular CHIPS were optionally sterilized by 10-min UV ozone treatment followed by overnight incubation in PSS at 37°C to remove residual gelatin.

### Bright-field and stereoscopic imaging

For all bright-field and stereoscopic images, we used a Leica M165FC microscope with a 1× Plan Apo lens, fully adjustable base with dark-field capability, and a Prime 95B complementary metal-oxide semiconductor (CMOS) camera. In addition, images of printed CHIPS and various equipment used were taken with either a Sony A5 camera equipped with a Laowa 24 mm f/14 probe lens, or an iPhone 14 pro. Image contrast adjustment and resizing was performed in FIJI ImageJ or Adobe Photoshop.

### OCT imaging and 3D gauging of CHIPS

The full 3D structure of CHIPS was imaged using OCT to noninvasively assess lumen patency for quality control and in-process monitoring as previously described (*64*). Briefly, OCT images were acquired using a Vega 1300 nm OCT system (Thorlabs, VEG210C1) mounted onto the bioprinter using an objective (OCT-LK4 objective) with an imaging depth of 11 mm and 13-μm lateral resolution. XYZ voxel sizes were acquired at 16.22 μm by 16.22 μm by 8.11 μm, respectively. Once scanned, XY, XZ, and YZ planes of the 3D images were analyzed to qualitatively assess the print for patency, significant defects, and potential blockages within the printed networks that would compromise flow through the CHIPS.

Quantification of perfusable channel diameter, filament diameter, infill spacing, and layer straightness from OCT images was performed in FIJI ImageJ using custom macros. To improve image contrast before channel diameter measurements and 3D gauging analysis, OCT images were despeckled, background subtracted, and thresholded to create a binary image. The image was inverted to have the channel lumens become bright signal. ROIs for a given CHIPS were selected using the rectangular selection tool, duplicated, and resliced to achieve single-pixel diameter representations. Analyze particles was then used to measure the width of the ROIs for >100 measurements per channel. Because of the tapered nature of many of the models, this quantification can induce nonspecific error due to the assumption of a constant diameter along the channel. For tapered networks, 3D gauging was performed.

Computational 3D gauging was performed to determine the deviation within the perfusable lumens between printed CHIPS and the original CAD design using a combination of FIJI ImageJ, Imaris (Bitplane v9.5), 3D Slicer, 3D builder, and Cloud Compare software. First, raw OCT images were denoised and scaled to account for RI of the imaging medium. Processed images were exported as TIFF stacks and imported to either Imaris or 3D slicer for segmentation of the internal perfusable networks (*69*). The segmented internal networks were exported as .STL files in 3D Slicer and imported into 3D builder to center the model at the origin. Both the segmented .STL and original .STL used for printing were imported into Cloud Compare 3D point cloud registration software. A standardized process was implemented to align the models to their bounding box centers and perform fine registration (*64*, *69*). A custom LUT centered on 0 was implemented to map positive (red, overprint) and negative (blue, underprint) deviations from the intended .STL onto the segmented .STL model. Gauging data viewed as both a histogram of error between the actual and CAD model and a 3D model with color coded deviation values were exported for further analysis, display, and graphing. The RMS error for each model was calculated from the overprint and underprint deviation results across all points within the compared volumes using Cloud Compare.

### Manual perfusion of branching vascular bed CHIPS

Perfusion of the branching vascular bed CHIPS was demonstrated by manually perfusing a concentrated ddH_2_O solution of blue food coloring through the top inlet of the construct with a 10-ml plastic BD syringe and a 20-gauge needle. The perfusion rate was modulated to achieve filling of the branching network and exit from the bottom outlet. Micromanipulators and helping hands devices were implemented to stabilize the perfusion without moving the printed construct. Perfusion was performed with the vascular bed construct submerged in a 50 mM Hepes buffer (pH 7.4).

### VAPOR assembly

VAPOR was designed with CAD software (Autodesk, Inventor; Autodesk, Fusion 360) and printed from Biomed Clear (Formlabs, RS-F2-BMCL-01) resin on a Form 3B (Formlabs, RS-F2-BMCL-01). Stainless steel M3 hex nuts (McMaster Carr, 94150A325) were inserted into the nut cutouts in the bioreactor main body. A glass coverslip was sealed into the lid by pipetting on 100-μl of resin followed by pressing a 22 mm–by–22 mm glass coverslip (VWR, 48366-227) into the lid, which was then sealed in place by baking in an UV oven at 60°C. Custom gaskets were cut from 1.5-mm-thick silicone sheeting (McMaster-Carr, 5787 T93) using a Cricut Maker and pressed into a cutout in the lid.

### Bioreactor perfusion system assembly

A 100-ml glass bottle (Cole Parmer, EW-34523-00) was used as a media reservoir. Holes for tubing lines were drilled into the cap, and 1/16″ (1.588 mm) ID silicone tubing (Cole Parmer, EW-95802-02) was pulled through to ensure airtightness. Two additional holes were drilled for air filtration and media exchange. A peristaltic pump (Ismatec, EW-95663-34) using 1.42 mm ID peristaltic tubing (Cole Parmer, EW-95663-34) was connected to the media reservoir tubing. Autoclavable bubble traps (Darwin Microfluidics, LVF-KBT-L-A) with 1/16″ (1.588 mm) barb adapters (Darwin Microfluidics, CIL-D-646) were placed after the pump and then connected to stopcocks on the reactor. Tubing exiting the reactor’s perfusion channels and lymph outlet returned to the media reservoir.

### Bioreactor perfusion of CHIPS

For sterile operation, 3D-printed parts were sonicated for 30 min in sterile filtered 70% ethanol, dried for 1 hour in a biosafety cabinet, and sterilized by 15-min UV ozone treatment. All remaining parts such as peristaltic tubing, media reservoir, and bubble traps were autoclaved. Alternatively, the entire perfusion system was preassembled and sterilized with ethylene oxide to simplify the workflow. For air filtration, a 0.2-μm pore size filter (VWR, 28145-501) was screwed onto the media reservoir. The system was assembled in a biosafety cabinet on an incubator shelf. All flow paths were purged at 1000 μl/min with media to avoid entrapment or perfusion of air bubbles in the tissue. CHIPS were then transferred into the VAPOR chamber. To transfer CHIPS into the system, the central well of VAPOR is first overfilled with sterile media or buffered solution. CHIPS are then scooped from their print container using a bent metal spatula and are transferred into the central well. Wide-tip forceps can also be used to gently pinch and lift CHIPS, whereas fine-tip forceps can apply too much local pressure and risk damaging the structure. CHIPS are then moved to align their inlets and outlets over VAPOR’s barbs. The excess water is aspirated to slowly lower CHIPS onto the barbs followed by applying gentle downward pressure to engage CHIPS inlets and outlets with the reactor barbs (Fig. 2, A_iii_). Sealing the lid then ensures that CHIPS remain secured against the barbs during perfusion. A constant rate of 100 μl/min was used for initial testing with visible dyes, fluorescent dextran diffusion studies, and perfusion of cellularized CHIPS. Higher flow rates of 1000 μl/min were used to validate leak-free performance. To maintain physiological temperature during cellularized perfusion, the system was then inserted into a 37°C incubator for perfusion culture. Cellularized CHIPS containing vascular bioink (HUVEC and MSC) were perfused with endothelial cell media, while pancreatic-like CHIPS were perfused with a 50:50 ratio of endothelial cell and MIN6 media.

### pH-sensitive dye perfusion

Serpentine CHIPS were placed in VAPOR reactors and perfused at 100 μl/min at each inlet. Colorimetric video acquisition was performed with a Sony A5 camera equipped with a Laowa 24 mm f/14 probe lens. During perfusion, one flow path consisted of an acidic phenol red solution adjusted to pH 6.5 using hydrochloric acid (HCl). The second flow path consisted of a basic PBS buffer solution adjusted to pH 11 using sodium hydroxide (NaOH). Initial perfusion was performed with a pulsatile roller pump (Masterflex 77202-60) to stimulate mixing along the serpentine network length. The change in phenol red color from the acidic yellow to basic magenta was quantified using the Color Profiler [ImageJ (*70*), downloaded and installed from https://imagej.net/ij/plugins/color-profiler.html] for a segmented line then traversed the length of the serpentine network. Values for magenta (white-green) and yellow (white-blue) were extrapolated from RGB intensity to determine the ratio of yellow:magenta along the path during different perfusion states. The ratio was graphed as a function of path length along the serpentine network and color coded to match the yellow and magenta values according to the pH indicator values for phenol red.

### Color dye perfusion

Laminar flow within serpentine CHIPS was demonstrated by perfusing either red or blue food coloring while maintaining equal flow rates of 100 μl/min in both channels. Vessel patency and dye diffusion into the bulk of stacked or 3D helical channel CHIPS was demonstrated by perfusing red and blue food coloring through separate channels. Colorimetric video acquisition was performed with a Sony A5 camera equipped with a Laowa 24 mm f/14 probe lens. Extended time lapse imaging was acquired with a GoPro Hero 5 camera mounted to a tripod.

### FITC-conjugated dextran perfusion

Dual parallel channel CHIPS were perfused at 100 μl/min with 3-, 10-, 40-, or 70-kDa dextran (0.1 mg/ml). Dextrans were conjugated with FITC (Thermo Fisher Scientific, D3305, D1821, D1844;,D1823). Dual parallel channel CHIPS’ second vessel was perfused with 1× PBS. CHIPS were perfused from 1 to 72 hours. Time lapse images were recorded on an epifluorescent stereomicroscope (either Nikon SMZ1000 or Leica M165FC) using an FITC filter, an X-Cite lamp (Excelitas), and a Prime 95B Scientific CMOS camera (Photometrics) with an image being taken every minute. To quantify dextran diffusion, fluorescence intensity over time was measured using ImageJ (National Institutes of Health) (*70*). Six ROIs were selected at increasing distances away from the FITC and PBS channels, and fluorescence intensity over time was calculated relative to the intensity at the initiation of perfusion while accounting for background signal.

### Microbead perfusion

Fluorescent polystyrene microbeads 10 μm in diameter were perfused at 100 μl/min at a concentration of 3.6 × 10^3^ beads/ml. The beads had either 580/605 (red) (Thermo Fisher Scientific, F8838) or 505/515 (yellow-green) (Thermo Fisher Scientific, F8836) excitation/emission wavelengths. The beads were perfused through dual parallel CHIPS in either the same or opposite directions, and videos were recorded on stereofluorescence microscopes with a GFO or TexasRed filter set similar to dextran perfusions. Particle tracking and bead velocimetry were performed in Imaris 9.5.1 (Bitplane) using spot detection and tracking algorithms.

### Perfusion of dual parallel channel CHIPS with afterload pressure

Dual parallel channel CHIPS were perfused as previously described at 100 μl/min with 40-kDa FITC-conjugated dextran (0.1 mg/ml) and 1× PBS. Each reservoir contained 20 ml of solution. Pressure within the CHIPS’ dextran channel was increased by raising the height of the dextran reservoir to produce an additional 5 or 10 mmHg of afterload. To assess the diffusion of dextran from the source channel into the systemic PBS circulation, 50 μl of samples was taken from the PBS reservoir bottle at 0 and 24 hours. The relative concentration of FITC-conjugated dextran compared to the source reservoir was then assessed by spectrophotometric analysis (Molecular Devices, SpectraMax i3x).

To assess the effect of afterload on molecular diffusion through CHIPS, time lapse images of perfusion with 5 mmHg of afterload (HP) were recorded as previously described and compared to perfusion with no additional afterload pressure (NP). The recordings were overlaid, and the fluorescence signal of the HP time lapse was divided by the NP time lapse after accounting for background signal. A vertical profile analysis was performed down the center of the HP/NP time lapse in ImageJ at various time points to further visualize the impact of increased afterload on diffusion into the peripheral regions of CHIPS.

### Parallel plate VAPOR perfusion

Parallel plate style CHIPS were perfused in a similar manner to CHIPS perfusion using an altered VAPOR layout to facilitate flow over an open-face sheet. A flow rate of 0.694 cm^3^/min was required to produce a shear rate of 0.1 dyne/cm^2^. Parallel plate style CHIPS were seeded with adult HDMECs (Lonza, CC-2543) in 25 μl volumes at 140,000 cells/cm^2^. Cells were allowed 24 hours to attach before being transferred into the parallel plate style VAPOR bioreactor to initiate flow. Samples were fixed after 10 days of perfusion for immunofluorescent staining and imaging.

### Multi-material needle alignment

To align multiple needles with micrometer accuracy, we created a custom dual camera optical alignment system (movie S5). Briefly, two 1×, 40-mm WD CompactTL Telecentric C-mount Lens (Edmund Optics, #63-745) were mounted to Alvium 1800 U-500 (Allied Vision) USB cameras. A custom 3D-printed alignment plate and XY positioning system allowed for focus adjustment to achieve parfocality. To image the bottom needle tip to obtain the XY position and needle diameter, a mirror (Thorlabs, ME2S-G01) was mounted at a 45° angle. The second camera was mounted perpendicular to the XY camera to view the side profile of the needle tip for Z-height alignment. A custom LabView program was written to simultaneously view the XY and Z positions. Each extruder was then moved to the center of the field of view for each camera to measure the relative XYZ offsets between each needle. The offset positions were stored as additional global software coordinate systems for use during multi-material printing using a custom script written within Aerotech CNC operator’s interface.

### Multi-material FRESH printing

3D models were prepared using Fusion 360 (Autodesk) for multi-material printing by creating individual nested components for each material within the desired location of the CHIPS. All components of multi-material CHIPS are available in a Zenodo repository (10.5281/zenodo.14975240). Each component was exported as an STL part file and imported into Cura 5.2 (Ultimaker) slicing software. The main components were centered on the XYZ origin and offset according to their designed spacing based on the original 3D model location. A separate material profile was created for each bioink within Cura to permit the assignment and indexing of the respective bioinks to one of the three extruders. In addition, the creation of individual bioink specific profiles enabled component-specific color visualization of the CHIPS within the slicing software. Custom start and end G-code was specified for each extruder tool profile to recall the stored position offsets determined during the alignment process and prime the extruder between tool changes.

### Immunofluorescence staining

Cellularized CHIPS were fixed via incubation in 10% neutral-buffered formalin (Sigma-Aldrich, HT501128) supplemented to a final molarity of 630 μM MgCl_2_ and 108 μM CaCl_2_. Tissues were then incubated in blocking buffer overnight. Blocking buffer consisted of 90% (v/v) 1× PBS supplemented to a final molarity of 1 mM CaCl_2_ and MgCl_2_ each, 5% (v/v) 1 M glycine (Thermo Fisher Scientific, BP381), 5% (v/v) goat serum (Thermo Fisher Scientific, 16210072), and 0.1% (v/v) Triton X-100 (Thermo Fisher Scientific, 85112). Samples were then immediately incubated with primary antibodies for a week at 4°C. Primary antibodies are diluted in antibody dilution buffer consisting of 1× PBS supplemented to a final molarity of 1 mM CaCl_2_ and MgCl_2_ each, 0.1% (w/v) bovine serum albumin (Sigma-Aldrich, A2153), and 0.1% (v/v) Triton X-100. Primary antibodies and their dilutions included VE-Cad rabbit monoclonal antibody (mAb, Cell Signaling Technology, 2500S) at 1:400, CD-31 mouse mAb (Cell Signaling Technology, 3528S) at 1:800, insulin mouse mAb (Cell Signaling Technology, 8138S) at 1:400, and insulin rabbit polyclonal (Abcam, ab181547) at 1:400. The samples were then washed three times for 1 hour in antibody buffer without Triton followed by an overnight wash at 4°C. The following day, the samples were incubated in secondary antibodies for a week at 4°C. All secondary antibodies were diluted in antibody dilution buffer. Secondary antibodies and their dilutions included 4′,6-diamidino-2-phenylindole (Sigma-Aldrich, D9542) at 1:400, phalloidin conjugated to Alexa Fluor 488 (Life Technologies, A12379) at 1:400, goat anti-rabbit immunoglobulin G (IgG) 555 (Thermo Fisher Scientific, A- 21428) at 1:1000 dilution, and goat anti-mouse IgG 633 (Thermo Fisher Scientific, A-21050) at 1:1000. The samples were then washed three times for 1 hour in antibody buffer without Triton followed by an overnight wash at 4°C.

### Tissue clearing

After immunofluorescent staining, the tissues were optically cleared using a 2:1 mixture of benzyl benzoate (Sigma-Aldrich) and benzyl alcohol (Sigma-Aldrich) (BABB). Samples were first serially dehydrated by 1 hour incubation each in 10, 25, 50, 75, 90, and 100% (v/v) ethanol solutions. The samples were then transferred into fresh 100% ethanol solution and incubated overnight at 4°C. Last, the samples were optically cleared by incubation in BABB for 1 to 24 hours before imaging depending on CHIPS thickness.

### Confocal imaging

All fluorescence confocal imaging was performed on a Nikon A1R HD MP multiphoton microscope equipped with a 4× [numerical aperture (NA) = 0.20] plan apochromat objective; 16× (NA = 0.80) long working distance water immersion objective; a 25× (NA = 1.10) plan apochromat water immersion objective; four visible light internal detectors; four visible laser lines (405, 488, 561, and 633 nm); and a motorized Prior Z-deck stage, Piezo Z Nosepiece, and Insight X3 DeepSee multiphoton laser (Spectra Physics). Large overview tile scans and 3D z-stack images of tissues were acquired using the 4× (NA = 0.20) plan apochromat (Nikon) objective with NIS Elements software. 3D rendering and image processing was performed in Imaris (v9.5, Bitplane). For cleared tissues, a custom-machined aluminum chamber was constructed to permit imaging through a large coverslip window and immobilization of the tissue within the BABB solution.

### Fluorescence image analysis

Advanced 3D fluorescence image analysis and animations were generated in Imaris 10.0 (Oxford Instruments). Specifically, the machine learning based vascular segmentation wizard was utilized to quantify the migratory network density and diameter within the vascular CHIPS. For quantitative measurements of deformation following perfusion and static culture of CHIPS, manual 3D measurements of infill spacing, filament diameter, channel length, width, and perimeter as well as CHIPS inlet/outlet spacing and overall scaffold dimensions were performed in Imaris 10.0. Colocalization analysis for the Actin channel with CD-31 channel in the pancreatic-like CHIPS was performed on ROI using the Coloc-2 plugin within FIJI Image-J. Pearson’s correlation coefficients and manders M1 and M2 values were recorded.

### Glucose-stimulated insulin secretion

GSIS was conducted using a static incubation approach in which FRESH bioprinted pancreatic-like CHIPS were removed from the bioreactor. The MIN6-containing pancreatic-like CHIPS were exposed to serial incubations in 4 ml of low-glucose (LG) (1.67 mM) and high-glucose (HG) (16.7 mM) Krebs buffer similar to previous work (*35*, *71*, *72*). A preincubation period in LG Krebs buffer was followed by serial incubations in fresh LG followed by HG Krebs buffer for 1.5 hours each. GSIS was performed in duplicate. Samples collected from each incubation phase were stored at −80°C for subsequent analysis of insulin concentration. Insulin content was analyzed using a mouse insulin ELISA (Mercodia) with each sample assayed in duplicate.

### Statistics and data analysis

Statistical analysis was performed with Prism 10 (Graphpad) using appropriate tests based on experimental conditions and data. For comparison of GSIS insulin concentrations, an unpaired *t* test was performed. Statistical significance was based on a *P < 0.05 with lower *P* values being denoted as **P < 0.01. Nonsignificant *P* values were denoted as ns.

Figures and visuals were constructed using Illustrator version 28.1 and Photoshop version 25.3.1 (Adobe). Supplementary videos and time-lapse images were edited in FIJI ImageJ and compiled in Premiere Pro version 24.1 (Adobe).
