# Supplementary material for: 3D bioprinting of collagen-based high-resolution internally perfusable scaffolds for engineering fully biologic tissue systems
Source: Sci Adv. 2025 Apr 23;11(17):eadu5905. doi: 10.1126/sciadv.adu5905 (PMC12017336; doi:10.1126/sciadv.adu5905)
Supplement: Supplementary file 1 — Figs. S1 to S13 Legends for movies S1 to S10 [file sciadv.adu5905_sm.pdf]

Science Advances
AAAS

Supplementary Materials for

3D bioprinting of collagen-based high-resolution internally perfusable scaffolds for engineering fully biologic tissue systems

Daniel J. Shiwarski *et al.*

Corresponding author: Daniel J. Shiwarski, djs87@pitt.edu; Adam W. Feinberg, feinberg@andrew.cmu.edu

Sci. Adv. **11**, eadu5905 (2025)
DOI: 10.1126/sciadv.adu5905

The PDF file includes:

Figs. S1 to S13
Legends for movies S1 to S10

Other Supplementary Material for this manuscript includes the following:

Movies S1 to S10

Supplementary Figures:

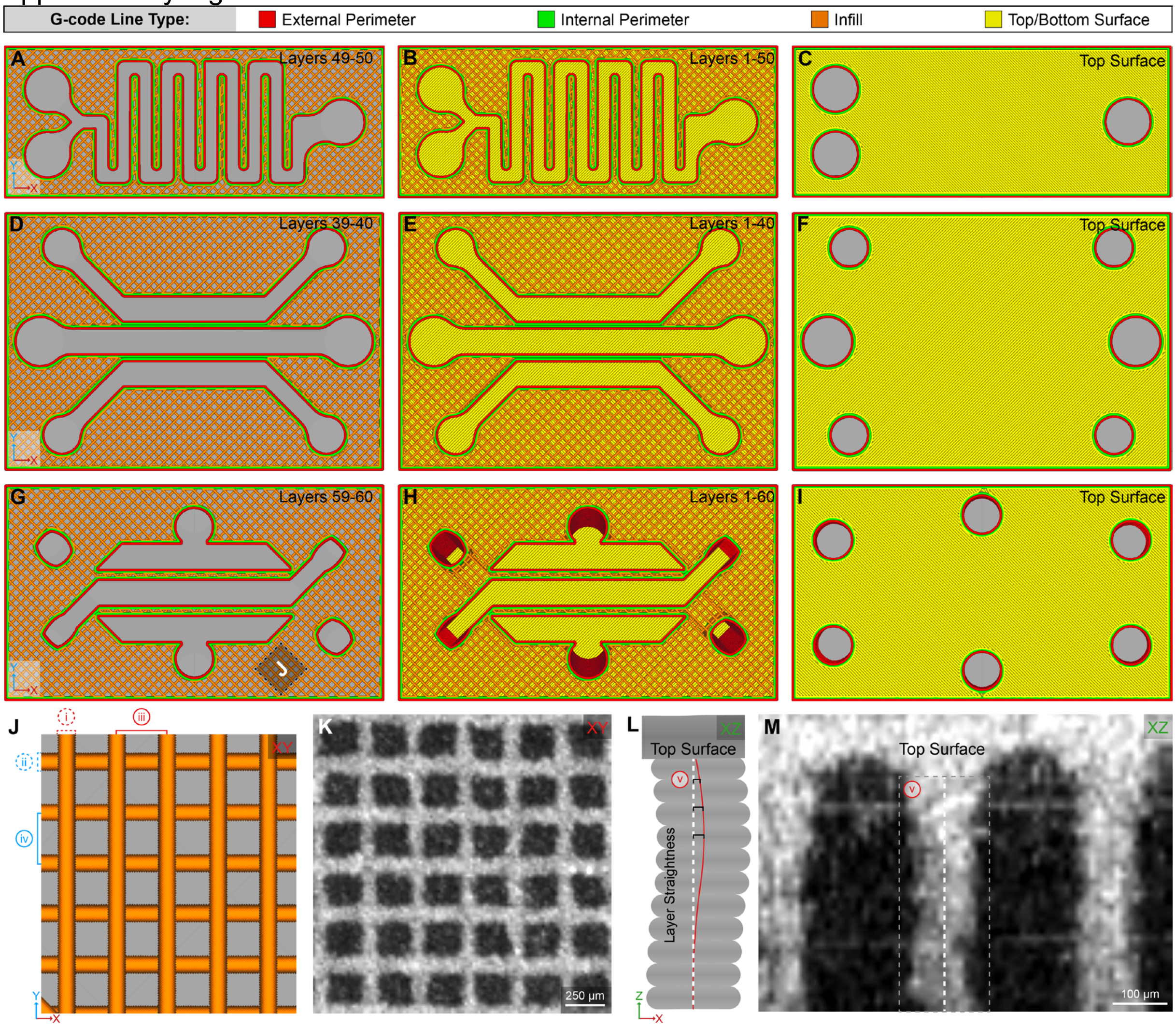

N	ⓘ	ⓘⓘ	ⓘⓘⓘ	ⓘⓥ	ⓥ
**Definition**	Filament Diameter$_x$	Filament Diameter$_y$	Infill Spacing$_x$	Infill Spacing$_x$	Layer Straightness$_{xz}$
**Expected** (µm)	90	90	301	301	0
**Measured** (µm)	108 ± 10 (n = 100)	103 ± 9 (n = 100)	308 ± 15 (n = 24)	313 ± 11 (n = 24)	6 ± 8 (n = 23)
**Error** (%)	20	15	2	4	-

Fig. S1. CHIPS G-code Renders and Quantification of XYZ Printing Fidelity. (**A** to **C**) 3D printing G-code renders of various layer views for the serpentine CHIPS highlighting external perimeters (red), internal perimeters (green), infill lines (orange), internal void space (gray), and bottom/top surfaces (yellow) that comprise the complete model. (**D** to **F**) G-code renders of various layer views for the triple channel CHIPS. (**G** to **I**) G-code renders of various layer views for the stacked channel CHIPS. (**J**) G-code section of infill from (G) used to define filament diameter$_{xy}$ in the x/y directions (i, ii) and infill spacing$_{xy}$ (iii, iv). (**K**) OCT imaging of internal infill region within layer 59 of the stacked CHIPS representing the corresponding region of interest depicted in (J). Extruded filaments shown in white with negative space shown in black. (**L**) Graphic illustration of the vertical layer straightness in the Z dimension achieved during the layer-by-layer printing process. Deviation (red line) from true straightness (dashed white line) is calculated to determine the average layer straightness within CHIPS. (**M**) OCT image of an XZ cross section through the top surface of the stacked CHIPS into the infill. Each vertical column (v) depicted represents approximately 20 stacked filament layers measured for their deviation from true straightness (dashed white line). (**N**) Table summarizing the quantification from (K) and (M) comparing the expected (G-code) to the printed and imaged (OCT) dimensions. All values are reported as mean ± std dev.

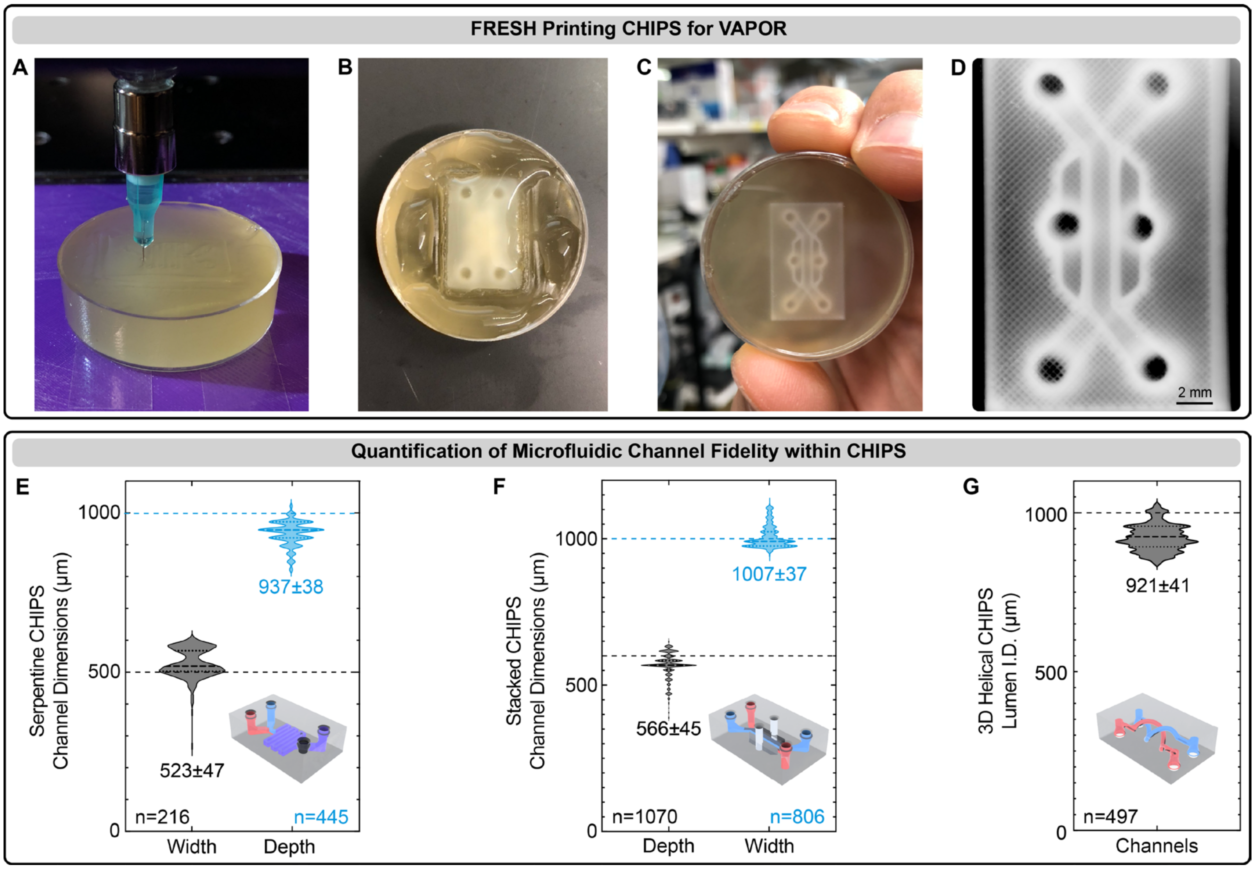

Fig. S2. FRESH printing CHIPS designed for VAPOR perfusion with internal network quantification. (**A**) A CHIPS model midway through FRESH printing within a gelatin support bath. (**B** and **C**) Top (B, 3D Helical) and bottom (C, Stacked) view of CHIPS immediately after FRESH printing. (**D**) A stereomicroscope image of a Stacked CHIPS model after release from the FRESH support bath. (**E** to **G**) OCT quantification of measured versus expected channel dimensions for Serpentine (E), Stacked (F), and 3D Helical (G) CHIPS. All values are reported as mean ± std dev across 2 or more CHIPS.

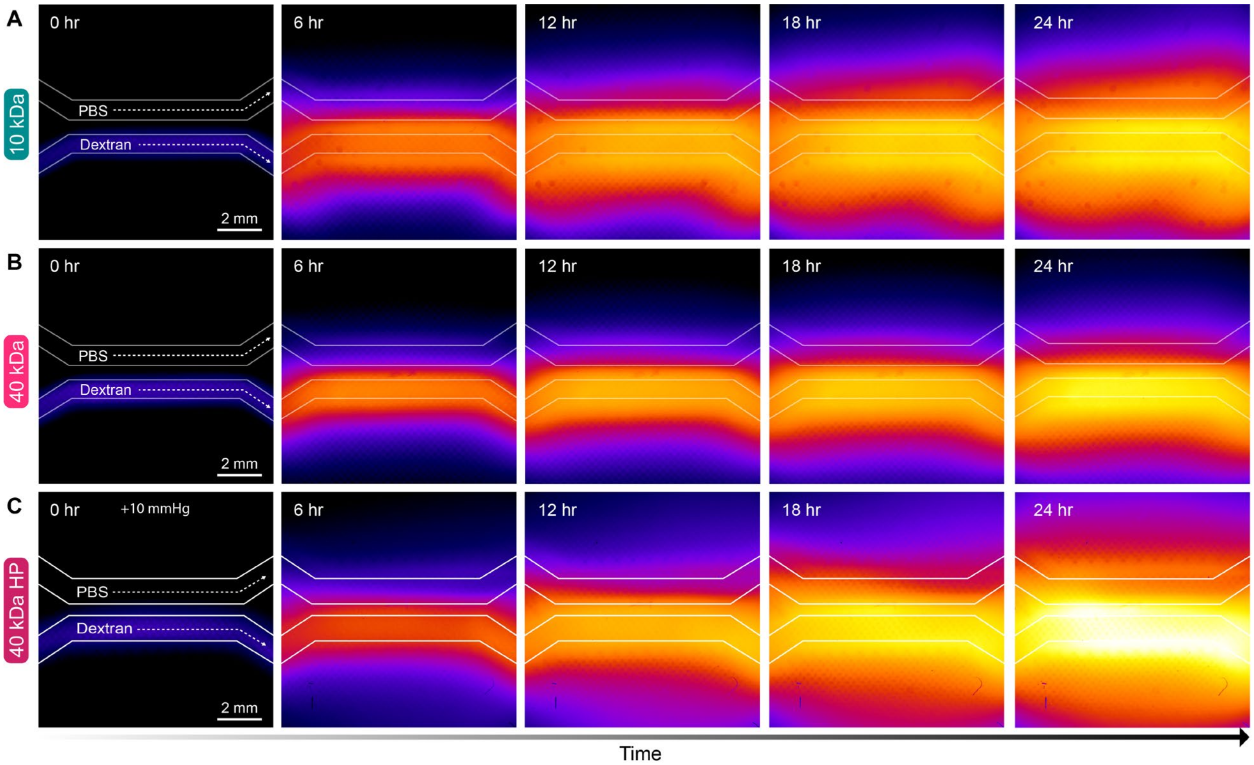

Fig. S3. Additional diffusivity analysis within dual channel CHIPS. (**A**) Time lapse images of dual parallel channel CHIPS undergoing VAPOR perfusion with FITC-conjugated 10 kDa dextran. (**B**) Time lapse images of dual parallel channel CHIPS undergoing VAPOR perfusion with FITC-conjugated 40 kDa dextran under normal pressure. (**C**) Time lapse images of dual parallel channel CHIPS undergoing high pressure (HP, +10 mmHg) VAPOR perfusion of 40 kDa FITC-conjugated dextran over 24 hours.

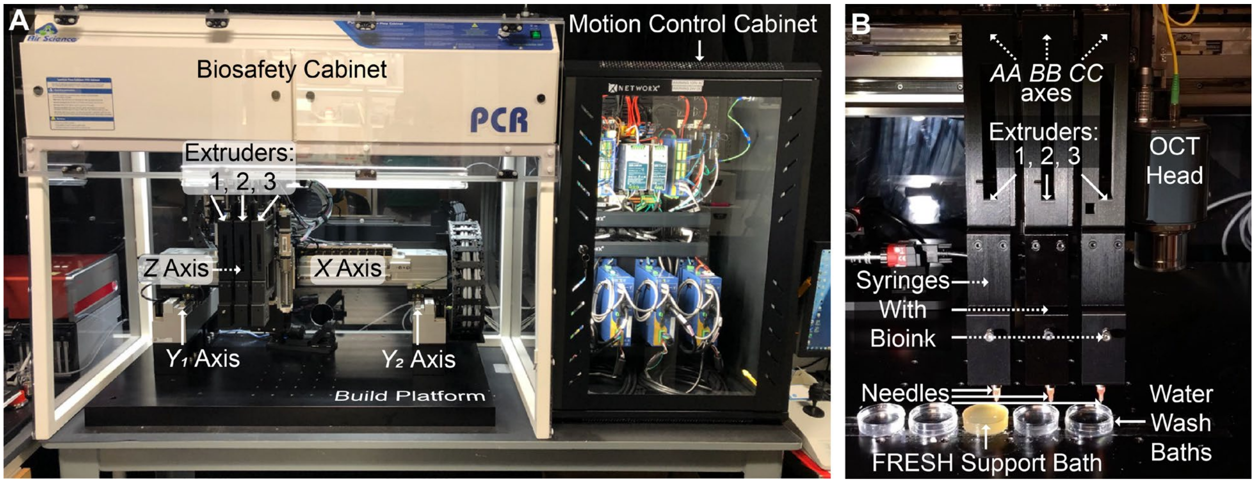

Fig. S4. Design and implementation of a multi-material bioprinter. (**A**) Custom high-performance 3D bioprinter based on a commercial Aerotech motion control platform and open-source Replistruder 5 syringe pumps. (**B**) Three Replistruder 5 syringe pumps are utilized for multi-material FRESH printing of CHIPS. An onboard OCT system allows for volumetric imaging and in-process monitoring of FRESH printed CHIPS.

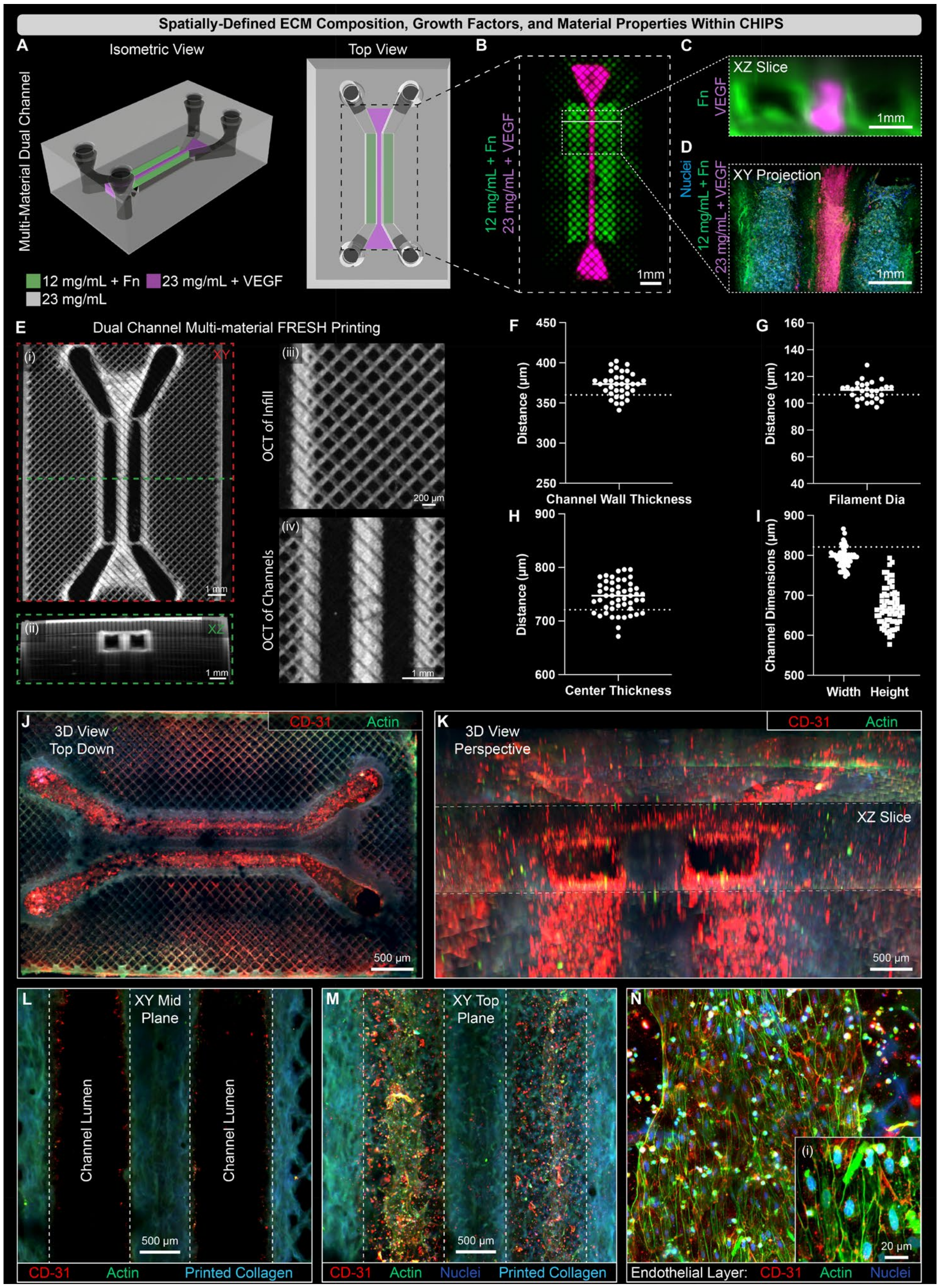

Fig. S5. Perfusion seeded endothelial cells form monolayers within multi-material CHIPS. (**A**) Dual channel CHIPS CAD design of internally lined channels containing 12 mg/mL collagen + Fn (green) with a central dividing region between the fluidic channels containing 23 mg/mL collagen + VEGF growth factor (magenta). (**B**) Confocal fluorescence maximum Z-projection image of the 12 mg/mL collagen channel lining and VEGF patterning. (**C**) XZ slice plane image from (B) demonstrating the Fn channel lining (green) and VEGF patterning (magenta). (**D**) Confocal fluorescence image of central channel regions from (B) seeded with HUVECs and stained for nuclei distribution (blue) at day 1 after seeding. (**E**) OCT cross-sectional views of multi-material CHIPS (A) with infill and channel regions used for fidelity measurements. (**F** to **I**) OCT quantification of measured versus expected dimensions of the channel wall thickness (F), infill filament diameter (G), center channel-dividing wall (H), and channel width and height (I) (mean plotted, $N \geq 30$ measurements each across 2 scaffolds, dashed line = expected value). (**J**) Volumetric confocal fluorescence imaging of optically cleared multi-material dual parallel channel CHIPS stained for endothelial cell marker CD-31 (red) and actin (green). (**K**) A 3D view with XZ cross-section of endothelial channel lining (red). (**L**) XY mid-plane view of open channel lumen and cell seeding. (**M**) An XY view of the channel top surface visualizing an endothelial monolayer coating the channel lumen. (**N**) Magnified XY images of endothelial lining within channels and increased zoom (i).

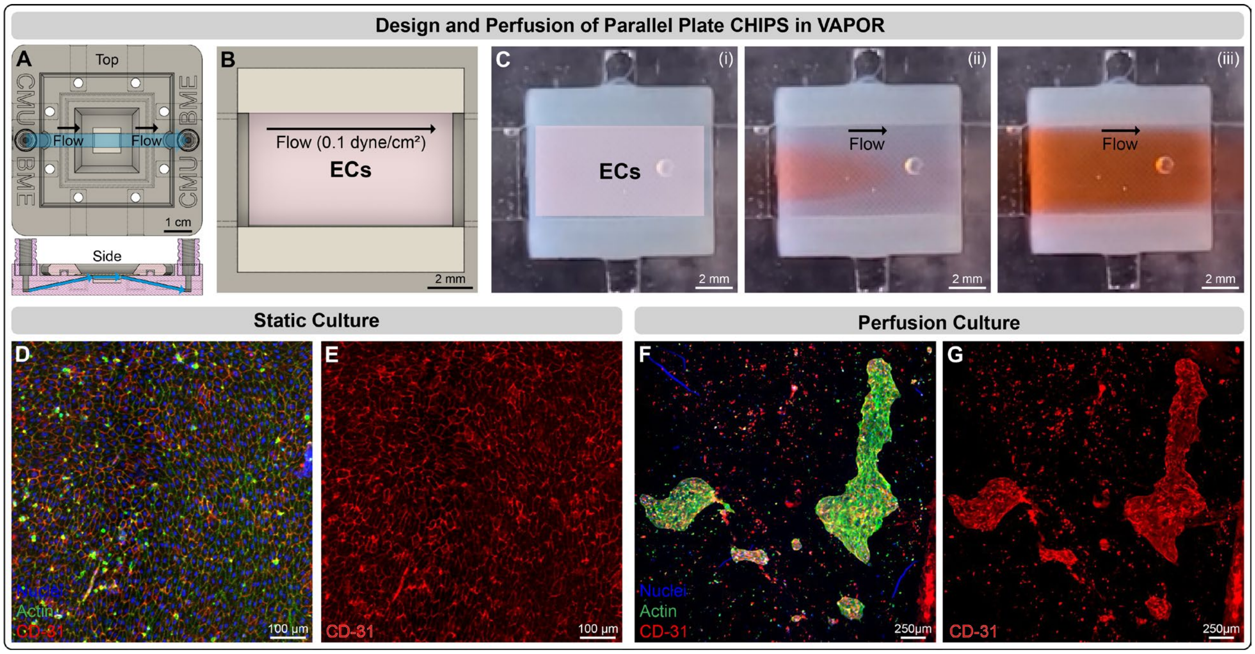

Fig. S6. Endothelial cell detachment under flow in parallel plate style CHIPS. (**A**) CAD model top and side views of the parallel plate CHIPS in VAPOR outlining the flow path (blue) of media over the CHIPS. (**B**) A CAD model top image of a parallel plate CHIPS seeded with ECs (pink) within the reactor. (**C**) Time lapse images perfusing parallel plate CHIPS with media (red) at 0.1 dyne/cm^2, demonstrating flow is contained to the cellularized region. (**D** to **G**) Immunofluorescence confocal images of ECs after 10 days of static culture (D and E) or perfusion culture (F and G).

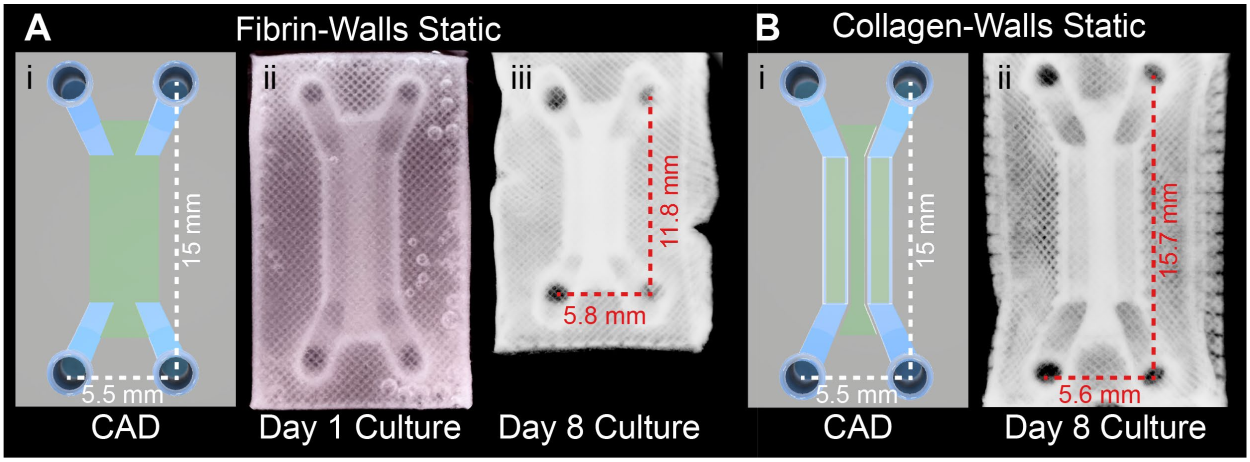

Fig. S7. The addition of collagen walls lining the perfusion channels prevent cell-driven buckling. (**A**) Dimensional comparison of the vascular CHIPS with complete cellular + fibrin walls CAD model (i) to the vascular CHIPS after 8-days of static culture (ii). **(B**) Dimensional comparison of the vascular CHIPS with collagen walls CAD model (i) to vascular CHIPS after 1 (ii) and 8-days of static culture (iii).

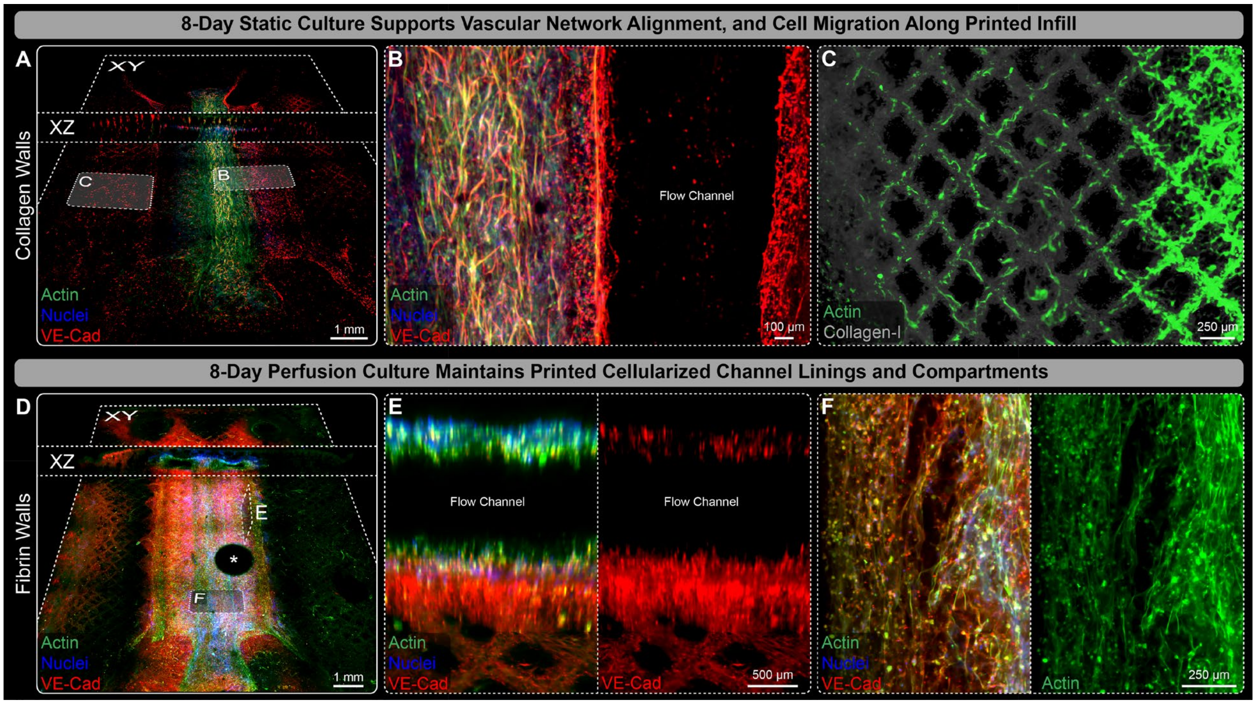

Fig. S8. FRESH printed cellularized channel linings and directed cell migration along printed collagen filaments within CHIPS. (**A**) Both XY and XZ perspective slice views of the channel bottom surface from 3D confocal imaging of optically cleared dual parallel channel cellular CHIPS with collagen walls statically cultured for 8 days. (**B** and **C**) Images reveal cellular alignment along the length of the channels, luminal VE-Cadherin expression (B), and evidence of cell migration following the printed collagen infill of distances exceeding 1.5 mm from the channel outside edge (C). (**D**) Both XY and XZ perspective midplane slice views from 3D confocal imaging of optically cleared dual parallel channel fibrin walls cellular CHIPS following 8 days of perfusion culture within VAPOR. (**E** and **F**) Images reveal enhanced VE-Cadherin expression around the flow channels (E), and extensive cell spreading throughout the central cellular region between channels (F). *Indicates air bubble artifacts introduced into the channels during CHIPS optical clearing and imaging.

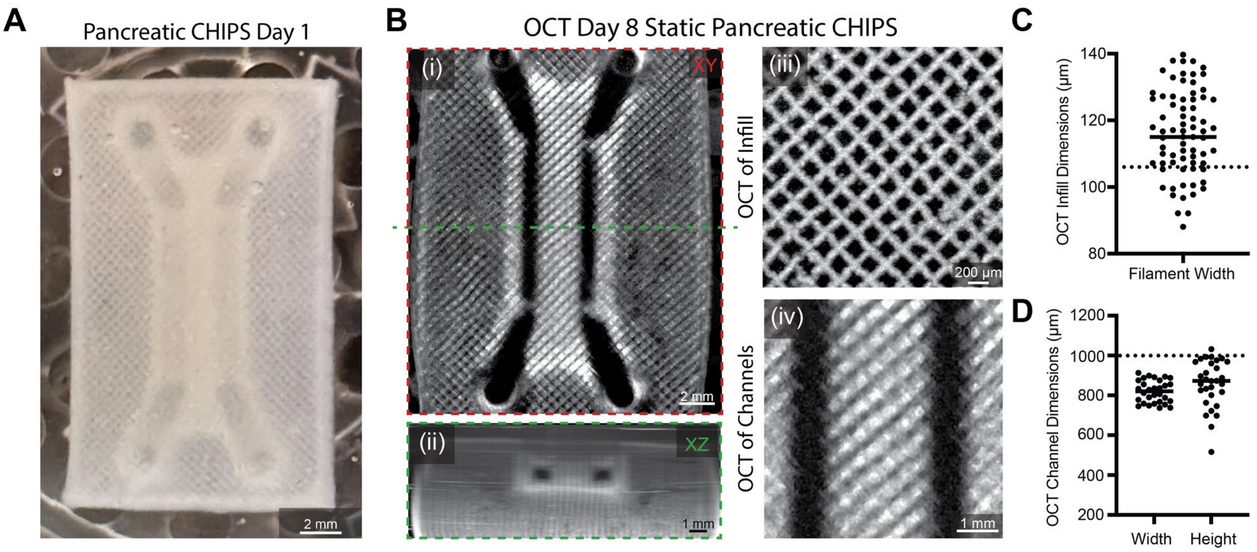

Fig. S9. Cellularized pancreatic-like CHIPS maintain geometric fidelity during static culture. (**A**) A pancreatic-like CHIPS at day 1. (**B**) OCT cross-sectional views of a pancreatic-like CHIPS after 8 days of static culture. (**C** and **D**) OCT quantification of measured versus expected dimensions of the infill filament diameter (C), and channel width and height (D) (mean plotted, $N \geq 30$ measurements each across 2 scaffolds, dashed line = expected value).

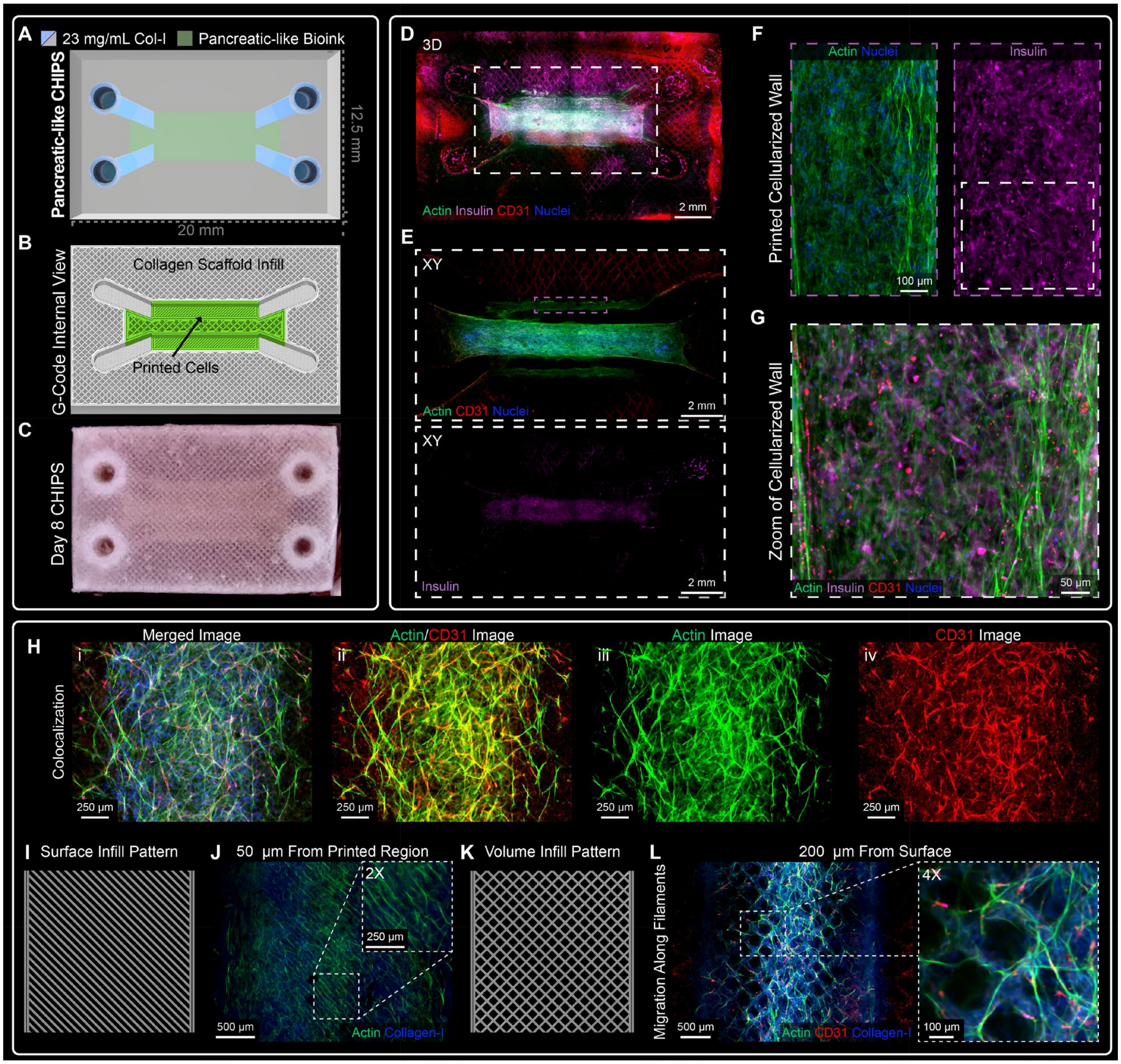

Fig. S10. Pancreatic-like CHIPS with fibrin walls express insulin and show a high degree of vascular-like migration along collagen filaments. (**A** and **B**) Schematic design and machine pathing G-code of pancreatic-like CHIPS with fibrin walls. (**C**) Stereomicroscope image of a pancreatic-like CHIPS after 8 days of static culture. (**D**) XY plane view from whole mount confocal fluorescence imaging of optically cleared 8-day statically cultured pancreatic-like CHIPS. (**E**) Zoomed region from (D) of the central cellularized area within the CHIPS. (**F**) Zoomed region of the printed channel wall from (E) highlighting the high cellularization achieved via 60 million cell/mL bioink and presence of insulin expression. (**G**) Zoom region from (F) showing the cellular distribution and protein expression within the fibrin printed wall after 8 days of static culture. (**H**) Confocal fluorescence imaging highlighting CD-31 colocalization with Actin within CHIPS resulting in a Pearson's Correlation Coefficient of 0.63 $\pm$ 0.04. (**I**) Graphic illustration of the FRESH printing pattern at the surface of the cellular region. (**J**) Confocal fluorescence image of the cellular alignment (actin, green) along the direction of the original print path (-45° angle) shown in (I). (**K**) Graphic illustration of the FRESH printed volume infill pattern (alternating $\pm$45° angle) at a depth 200 µm from the surface of the cellular region. (**L**) Cellular migration (actin, green) along the printed collagen filaments (blue) 200 µm below the original printed cellular region.

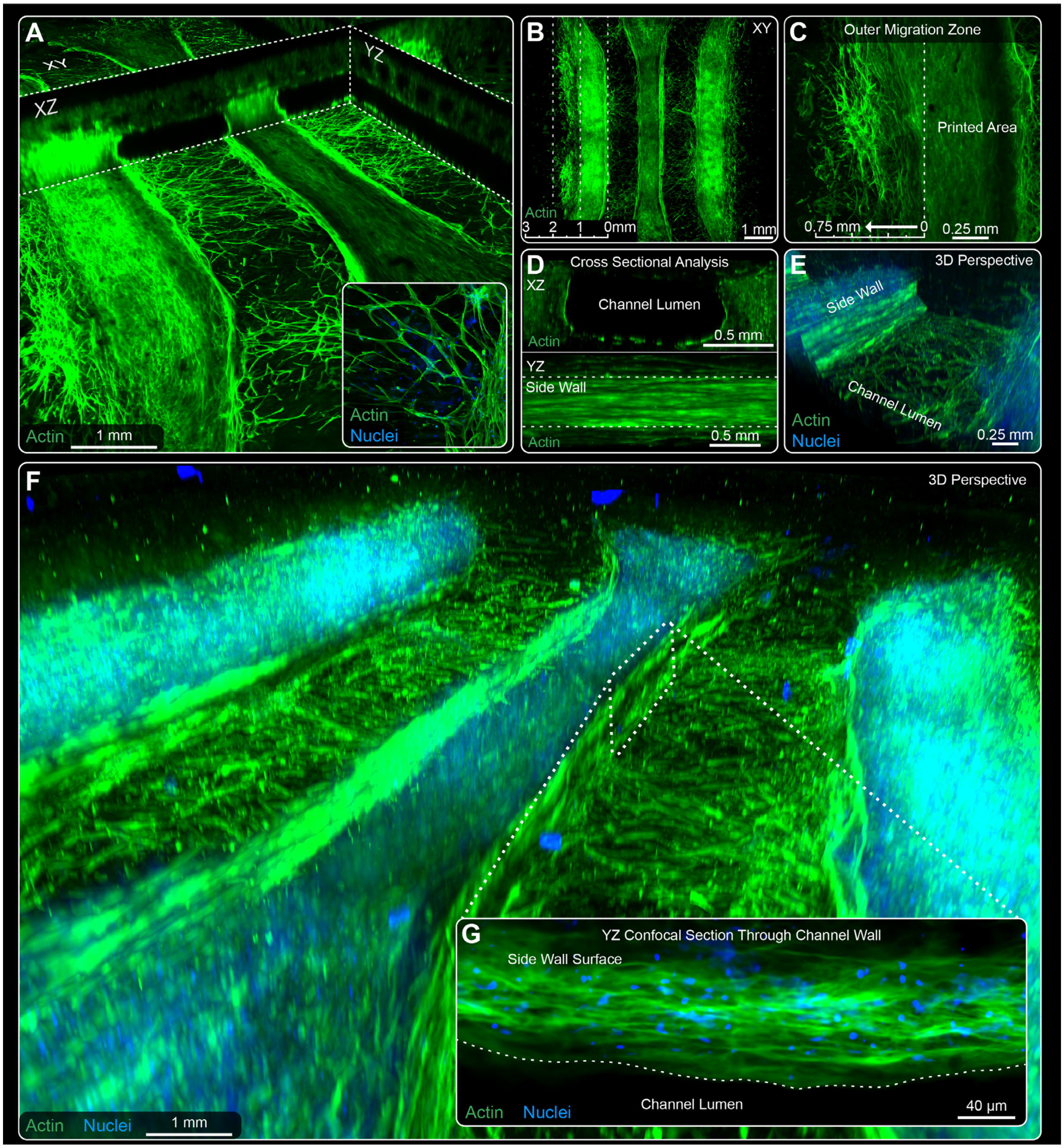

Fig. S11. Highly migratory and branching cellular networks within statically cultured pancreatic-like CHIPS. (**A**) Confocal fluorescence images of XY, YZ, and XZ plane views revealing the intricate cell network and migration between the patent flow channels. (**B**) XY max intensity Z-projection image demonstrating the range of cell migration (actin, green) outward from the printed regions. (**C**) Zoom in image to the outer migration zone. (**D**) XZ cross sectional analysis expression profile of the cellular (actin, green) markers lining the original acellular channels, in addition to a YZ projection showing a dense luminal cell monolayer along the side walls. (**E** and **F**) Isometric views of cell migration into the channel lumen. (**G**) A YZ projection through the channel wall.

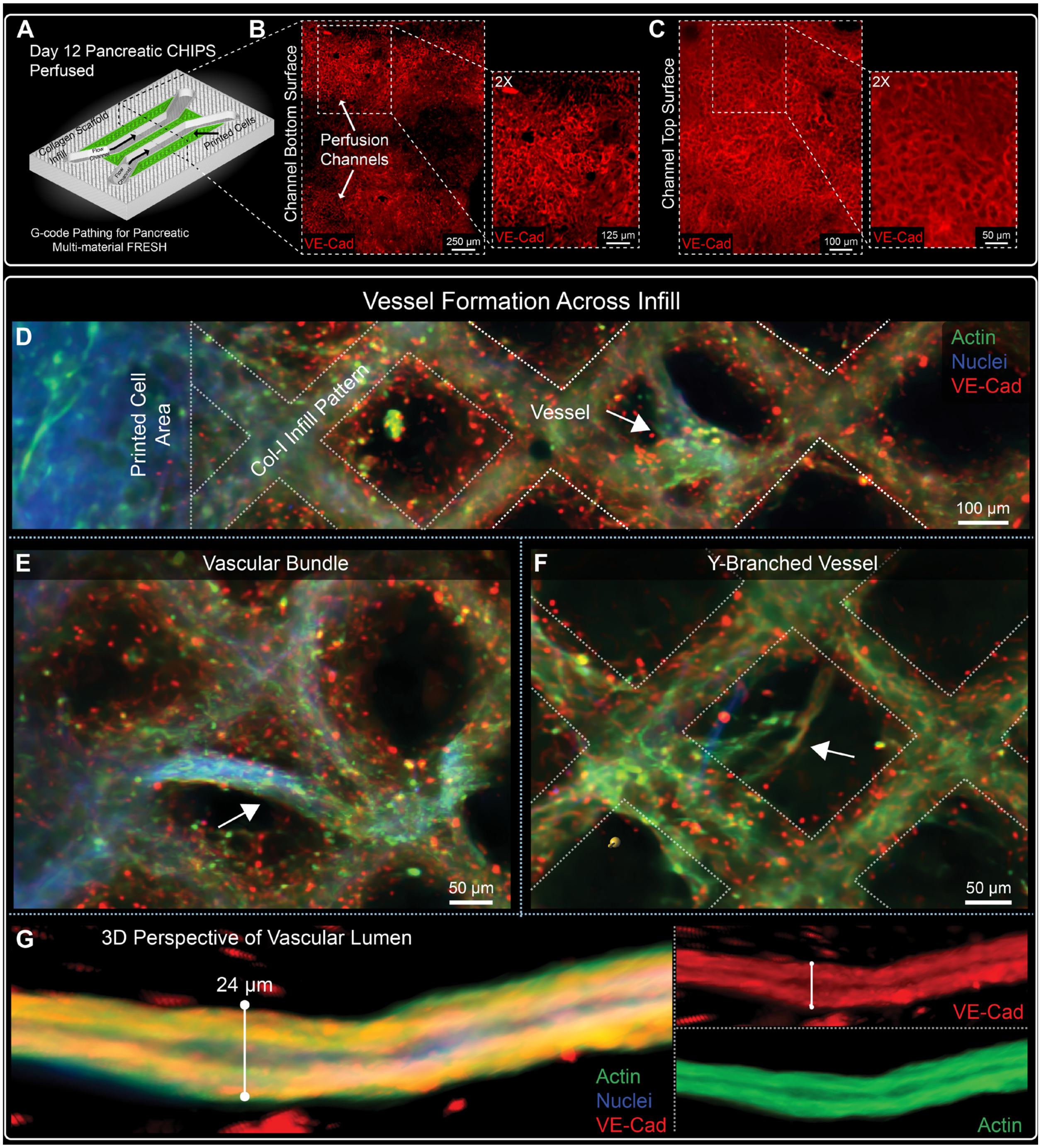

Fig. S12: Vasculogenesis and formation of branching networks across printed infill patterns. (**A**) The 3D printer machine pathing G-code of a pancreatic-like CHIPS. (**B** and **C**) Confocal images of the channel's bottom (B) and top (C) surfaces with regions of VE-Cad^{+} cell monolayers on the originally acellular channels. (**D** to **F**) Confocal fluorescence images from Fig. 7E demonstrating a (D) branched vessel-like structure 100 µm in diameter, (E) A vascular-like bundle of 50 µm in diameter bridging and following the infill lattice structure, and (F) a 25 µm Y-branched vascular-like structure bridging across the collagen infill pattern (outline with dotted white lines) within the CHIPS. (**G**) 3D perspective view of a 24 µm vessel with visible open lumen expressing actin (green) and VE-cadherin (red).

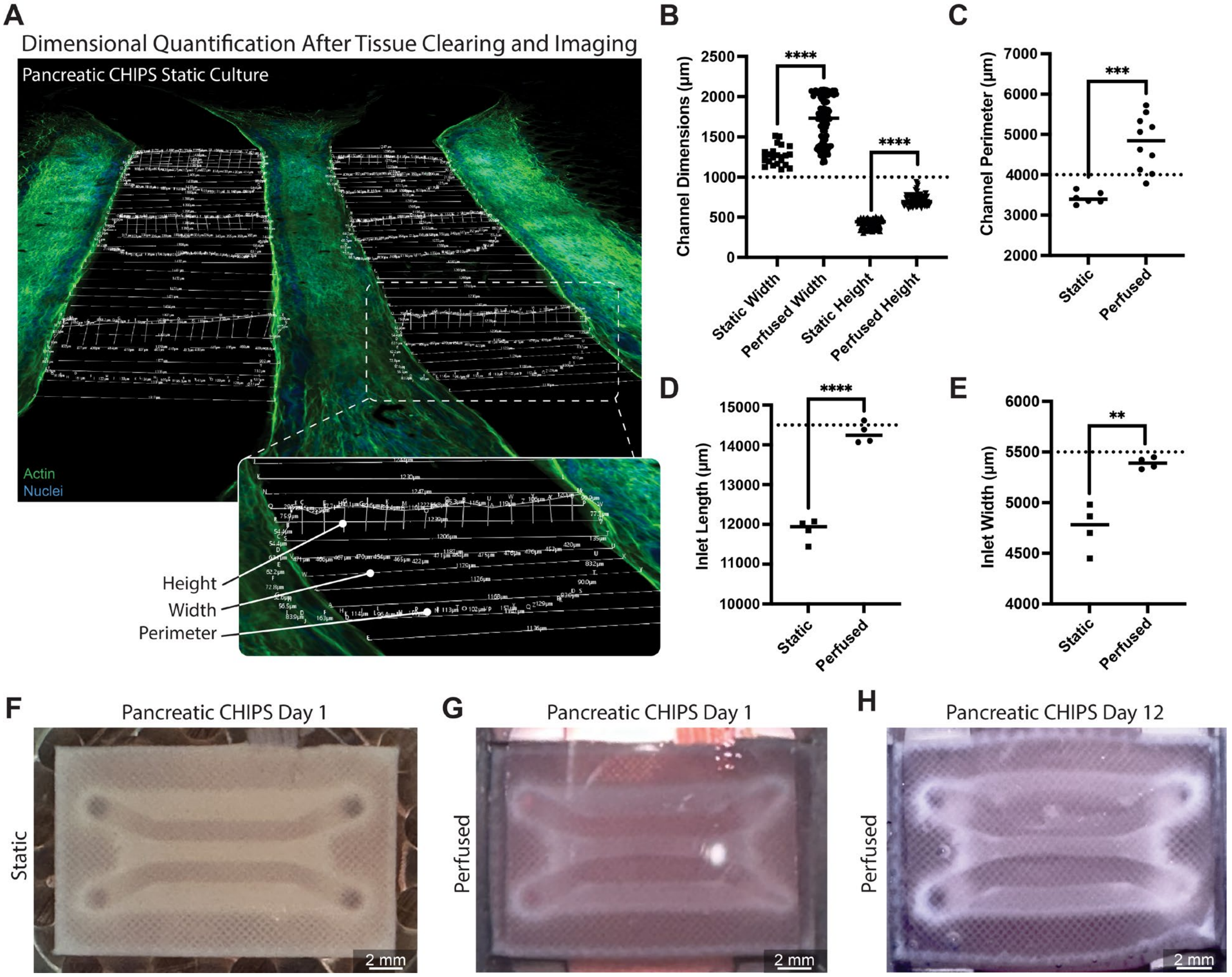

Figure S13: Cellularized pancreatic-like CHIPS maintain geometric fidelity during perfusion culture. (**A**) An example of dimensional quantification of a static culture pancreatic-like CHIPS' channels. (**B** to **E**) Quantification of measured versus expected dimensions for channel width and height (B), perimeter (C), inlet length (D) and inlet width (E) (mean plotted with all replicates, $N = 2$ CHIPS across repeated measurements when applicable, $**P < 0.01$, $***P < 0.001$, $****P < 0.0001$, unpaired t-test between static and perfused conditions, dashed line = expected value based on CAD design). (**F** to **H**) Brightfield images of pancreatic-like CHIPS at day 1 of static culture (F), and after 1 day (G) or 12 days (H) of perfusion culture within VAPOR.

Captions for Movies S1 to S10

Movie S1. Branching Vascular Bed CHIPS Fabrication. Visualization of the CAD model render, G-code print pathing, and OCT imaging for each slice of the branching vascular bed CHIPS as the model is 3D printed from bottom to top. Dark void space within the OCT image reveals the open channel network and white signal highlights the printed collagen filaments. Example printing simulation shows the actual path and travel moves of the print head and order of printing during layer 25 of the model. External perimeter displayed in red, interior perimeter displayed in green, and infill pattern displayed in orange. Final image displays the completed FRESH printed CHIPS followed by the manual perfusion of blue dye through the branching vascular bed.

Movie S2. VAPOR Assembly and Serpentine Network Perfusion Demonstrating Laminar Flow and Fluid Mixing. 3D render and animation of the VAPOR assembly with a serpentine CHIPS followed by the perfusion of a red and blue dye utilizing laminar flow. A mixing gradient was established via pulsatile perfusion of an acidic phenol red solution (yellow) and a basic NaOH solution.

Movie S3. OCT Validation and VAPOR Perfusion of Stacked and 3D Helical CHIPS. XY and XZ slice views from the 3D OCT image of the stacked CHIPS revealing network patency and high-fidelity printing followed by red and blue dye perfusion using VAPOR. XY, XZ, and YZ slice views from the 3D OCT image of the helical CHIPS demonstrating the channel circularity and spiral networking within the collagen CHIPS. Perfusion of red and blue dye over a 24-hour period using VAPOR highlights the inherent diffusivity within CHIPS due to the microporosity of FRESH-printed collagen.

Movie S4. Fluorescence Bead and Dextran Perfusion of Dual Channel CHIPS with VAPOR Showing Molecular Weight and Pressure Dependance. Video of fluorescence microbeads demonstrates the integrity of the FRESH-printed dual channel CHIPS and the laminar flow profile achieved via particle tracking. Additionally, perfusion of 3, 10, 40, and 70 kDa fluorescent dextrans show a molecular weight dependance on the rate of diffusion through the CHIPS. Fluorescence intensity has been pseudo-colored with the fire LUT to highlight the diffusion gradient. By increasing the pressure of the dextran perfusate we show the dextran can diffuse more rapidly into distant regions of the CHIPS displayed as the ratio of high-pressure images to normal pressures images and pseudo-colored with the jet LUT.

Movie S5. Example 3D Bioprinting Set-up and Needle Alignment for Multi-material Printing. Overview of the Aerotech-based custom 3D bioprinting and multi-material alignment set-up for needle registration. Video demonstrates switching between materials and printing of 3-material CHIPS into a FRESH support bath. 3D confocal fluorescence images highlight the accuracy of multi-material printing single layer and branching parallel plate style CHIPS.

Movie S6. 3D Perspective and Flythrough of Multi-material Dual Channel CHIPS. Confocal fluorescence imaging of optically-cleared dual channel CHIPS shows a high degree of internal multi-material alignment for collagen and growth-factor laden biomaterials within a CHIPS seeded with HUVECs.

Movie S7. 3D Multi-material Cell Printing and Flythrough of FRESH-printed Vascular-like CHIPS. Confocal fluorescence imaging of optically-cleared dual channel vascular CHIPS after

printing demonstrates high volumetric registration between the fibrin-based cell ink and the collagen frame. Confocal fluorescence imaging of optically-cleared vascular CHIPS after 8-days in static culture demonstrates high cell density and prominent VE-cadherin expression throughout a vascular-like network within the volume of the vascular bioink printed regions for both the fibrin walls and collagen reinforced walls CHIPS. Additionally, perfusion of the vascular CHIPS with VAPOR promotes vessel-like changes in cell morphology and enhances the VE-cadherin expression around the perfusable channels.

Movie S8. Statically cultured MIN6-Containing Pancreatic-like CHIPS Exhibit Dense Vascular-like Network Formation and Cell Migration within the Scaffold. Following 12-days of static culture and confocal fluorescence imaging of the optically-cleared pancreatic-like CHIPS, we observed branching vascular-like network formation and extensive cell migration into the surrounding collagen scaffold.

Movie S9. VAPOR Perfused MIN6-Containing Pancreatic-like CHIPS Exhibit Vessel-like Branching Across Collagen Infill of the Scaffold. Example confocal z-stacks from regions below the perfusable channels within the CHIPS show cellular structures emanating from the printed regions that follow the printed collagen infill and branch across the open space in both single and Y-branching VE-cadherin$^+$ vessel-like structures.

Movie S10. Dense Pancreatic-like Microlumen Formation in VAPOR Perfused MIN6-Containing Pancreatic-like CHIPS. Following 12-days of perfusion culture in VAPOR, the optically-cleared pancreatic-like CHIPS exhibit enhanced insulin expression and morphological changes within insulin$^+$ structures that resemble early pancreatic developmental stages such as budding and microlumen formation.
